# Downregulation of UHRF1 increases tumor malignancy by activating the CXCR4/AKT-JNK/IL-6/Snail signaling axis in hepatocellular carcinoma cells

**DOI:** 10.1038/s41598-017-02935-2

**Published:** 2017-06-05

**Authors:** Ji-Hyun Kim, Jae-Woong Shim, Da-Young Eum, Sung Dae Kim, Si Ho Choi, Kwangmo Yang, Kyu Heo, Moon-Taek Park

**Affiliations:** Research Center, Dongnam Institute of Radiological & Medical Sciences (DIRAMS), Busan, 46033 Republic of Korea

## Abstract

UHRF1 (ubiquitin-like, with PHD and RING finger domains 1) plays a crucial role in DNA methylation, chromatin remodeling and gene expression and is aberrantly upregulated in various types of human cancers. However, the precise role of UHRF1 in cancer remains controversial. In this study, we observed that hypoxia-induced downregulation of UHRF1 contributes to the induction of the epithelial-mesenchymal transition (EMT) in hepatocellular carcinoma cells. By negatively modulating UHRF1 expression, we further showed that UHRF1 deficiency in itself is sufficient to increase the migratory and invasive properties of cells via inducing EMT, increasing the tumorigenic capacity of cells and leading to the expansion of cancer stem-like cells. Epigenetic changes caused by UHRF1 deficiency triggered the upregulation of CXCR4, thereby activating AKT and JNK to increase the expression and secretion of IL-6. In addition, IL-6 readily activated the JAK/STAT3/Snail signaling axis, which subsequently contributed to UHRF1 deficiency-induced EMT. Our results collectively demonstrate that UHRF1 deficiency may play a pivotal role in the malignant alteration of cancer cells.

## Introduction

UHRF1 (ubiquitin-like with PHD (plant homeodomain) and RING (Really Interesting New Gene) finger domains 1) contributes to the maintenance of DNA methylation by recruiting DNMT1 to hemimethylated DNA, thereby ensuring that the DNA methylation patterns of mother cells are correctly imparted to daughter cells^1^. UHRF1 is a multi-domain protein that contains an N-terminal ubiquitin-like domain, a tandem tudor domain, a PHD domain, an SRA domain and a RING finger motif-domain^2^. Its PHD and SRA domains are responsible for its interaction with DNMT1 and hemimethylated DNA^2^. In particular, UHRF1 is known as an E3-ubiquitin-ligase for DNMT1 because the RING finger motif of UHRF1 has an E3-ubiquitin-liagase function^2, 3^. Due to this property, UHRF1 upregulation can lead to the global DNA hypomethylation, a hallmark of cancer^2, 3^. In addition, because UHRF1 is upregulated in many types of cancer cells, it has been considered an oncogene or a prognostic marker for cancer patients^4^. Interestingly, disruption of the PCNA/DNMT1/UHRF1 complex induces global DNA hypomethylation and oncogenic transformation. Furthermore, global DNA hypomethylation can also occur through UHRF1 deficiency^5, 6^. However, the precise mechanism by which UHRF1 deficiency contributes to cancer progression has not yet been elucidated.

Hepatocellular carcinoma is widely known to be one of the most aggressive diseases due to its poor prognosis and high recurrence rate caused by metastasis, which is associated with the epithelial-mesenchymal transition (EMT)^7, 8^. A highly conserved cellular process, EMT plays a pivotal role in tumor malignancy^8, 9^.

In that regard, the expression of epithelial markers is decreased during the EMT process, whereas the expression of mesenchymal markers is increased^10, 11^. These alterations lead to impaired cell-cell adhesion, consequently allowing the dissemination of cancer cells from primary sites to distant secondary sites^12, 13^. In addition, EMT is recognized as a potential mechanism for the generation of cancer stem-like cells known to be responsible for tumor initiation, metastasis, recurrence and resistance to chemo- and radiotherapy^14, 15^. Due to these properties of cancer stem-like cells, targeting them has recently been deemed a key strategy for cancer therapeutics^15, 16^.

Many cytokines and their receptors regulate tumor progression^17, 18^. In particular, the signaling axis activated by stromal-derived growth factor-1 (SDF1, also described as CXCL12) and its receptor CXCR4 can influence metastatic spread in diverse tumor types^19–21^. Furthermore, CXCR4 overexpression highly correlates with aggressiveness and poor prognosis^19, 22^. Additionally, CXCR4 is thought to be a candidate marker for cancer stem-like cells and has a fundamental role in the maintenance and growth of cancer stem-like cells *in vitro* and *in vivo* ^23, 24^.

Previously, we found that the downregulation of UHRF1 leads to the induction of EMT via CXCR4 in human cancer cells^25^. In this current study, we further investigated the signaling mechanisms underlying the role of UHRF1 deficiency in EMT associated with tumor malignancy. Our present data demonstrate that hypoxia decreases the level of UHRF1, thereby increasing EMT. Additionally, the activation of the CXCR4/AKT-JNK/IL-6 signaling axis is required for UHRF1 deficiency-induced EMT. Our findings provide a novel mechanism that may explain how UHRF1 deficiency is positively associated with tumor malignancy.

## Results

### UHRF1 deficiency promotes mesenchymal transformation via activating EMT

**Figure 1:**
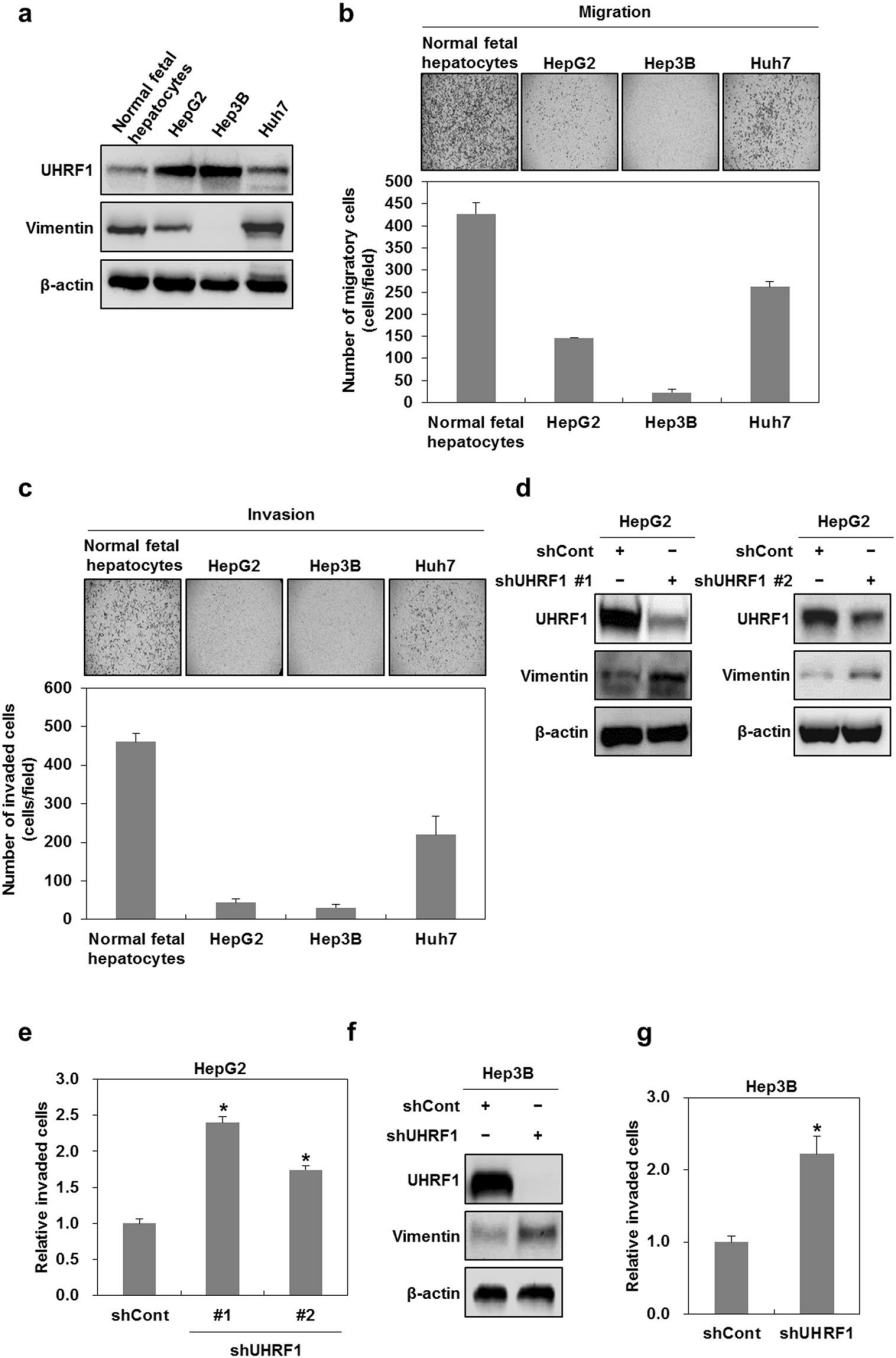
Downregulation of UHRF1 enhances the migratory and invasive properties of hepatocellular carcinoma cells. (**a**) Western blot analysis for UHRF1 and vimentin in normal fetal hepatocytes, HepG2, Hep3B and Huh7 cells. (**b** and **c**) Comparisons between the migratory and invasive properties of normal fetal hepatocytes, HepG2, Hep3B and Huh7 cells. The migration and invasion assay were performed using the Transwell chamber. (**d**) Western blot analysis for UHRF1 and vimentin in shCont-, shUHRF1#1- and shUHRF1#2-HepG2 cells. (**e**) A comparison of invasive properties in shUHRF1#1-, shUHRF1#2-HepG2 and HepG2 cells transiently transfected with siRNA targeting UHRF1. The invasion assay was performed using the Transwell chamber. (**f**) Western blot analysis for UHRF1 and vimentin in shCont- and shUHRF1#1-Hep3B cells. (**g**) Effect of shRNA targeting UHRF1 on the invasive properties of Hep3B cells. The invasion assay was performed using the Transwell chamber. β-actin was used as a loading control. Results from three independent experiments are expressed as means ± SEMs. (*P < 0.05, **P < 0.01).

To validate our previous report^25^, we investigated whether UHRF1 deficiency is associated with the acquisition of mesenchymal characteristics in hepatocellular carcinoma cells. In this regard, we performed western blot analysis to compare the basal levels of UHRF1 and the mesenchymal marker vimentin in HepG2, Hep3B and Huh7 cells with that in normal human fetal hepatocytes (HFHs). Although the basal level of UHRF1 in these cancer cells was higher than that in HFHs, it was greatly reduced in Huh7 cells compared with the other cancer cells (Fig. 1a). However, the basal level of vimentin was highly increased in HFHs and Huh7 compared with that in HepG2 and Hep3B cells, which have relatively higher levels of UHRF1. The ability of HFHs and Huh7 cells to migrate and invade was also substantially greater than that of HepG2 and Hep3B cells, indicating that the downregulation of UHRF1 may be related to an increase in cell migratory/invasive ability and processes that are involved in EMT (Fig. 1a and b). In addition, HFHs have features similar to hepatic progenitor cells, which possess self-renewal capacity and migratory potential^26^. Thus, compared with these cancer cells, the relative increase in the migratory and invasive abilities of HFHs is thought to be due to these reported characteristics.

To examine whether UHRF1 downregulation has a positive influence on EMT, we introduced shRNAs targeting UHRF1 (shUHRF1#1 or shUHRF#2) into HepG2 cells, which expresses high levels of UHRF1, and generated stable UHRF1-deficient HepG2 cells. Thereafter, we performed western blot analysis to measure the mesenchymal marker vimentin. As shown in Fig. 1d, we found an increase in the level of vimentin in UHRF1-deficient HepG2 cells compared with parental cells. When we confirmed this result using transient transfection with siRNA targeting UHRF1, we obtained similar results to those obtained using stable cells (Supplementary Fig. S1a). Consistent with the increased level of vimentin, both shRNAs and siRNAs targeting UHRF1 effectively increased the invasiveness of HepG2 cells (Fig. 1e and Supplementary Fig. S1b). Therefore, we investigated most of the cellular and molecular events concerned with EMT using shUHRF1 #1 HepG2 cells. In addition, when we confirmed these events in Hep3B cells stably transfected with shUHRF1#1, we also obtained similar results to shUHRF1 #1 HepG2 cells (Fig. 1f and g).

We next investigated whether UHRF1 affects the high migratory and invasive abilities of Huh7 or HFHs, which have relatively lower levels of UHRF1. As shown in Supplementary Fig. S1c and d, UHRF1 overexpression suppressed the expression level of vimentin in both Huh7 cells and HFHs. Furthermore, we found that UHRF1 overexpression effectively inhibited the migration and invasiveness in both these cells, suggesting that UHRF1 can contribute to suppressing the EMT of the cells (Supplementary Fig. S1e and f).

Collectively, these results indicated that UHRF1 downregulation leads to mesenchymal transformation via activating EMT.

### Hypoxia leads to UHRF1 downregulation in hepatocellular carcinoma cells

**Figure 2:**
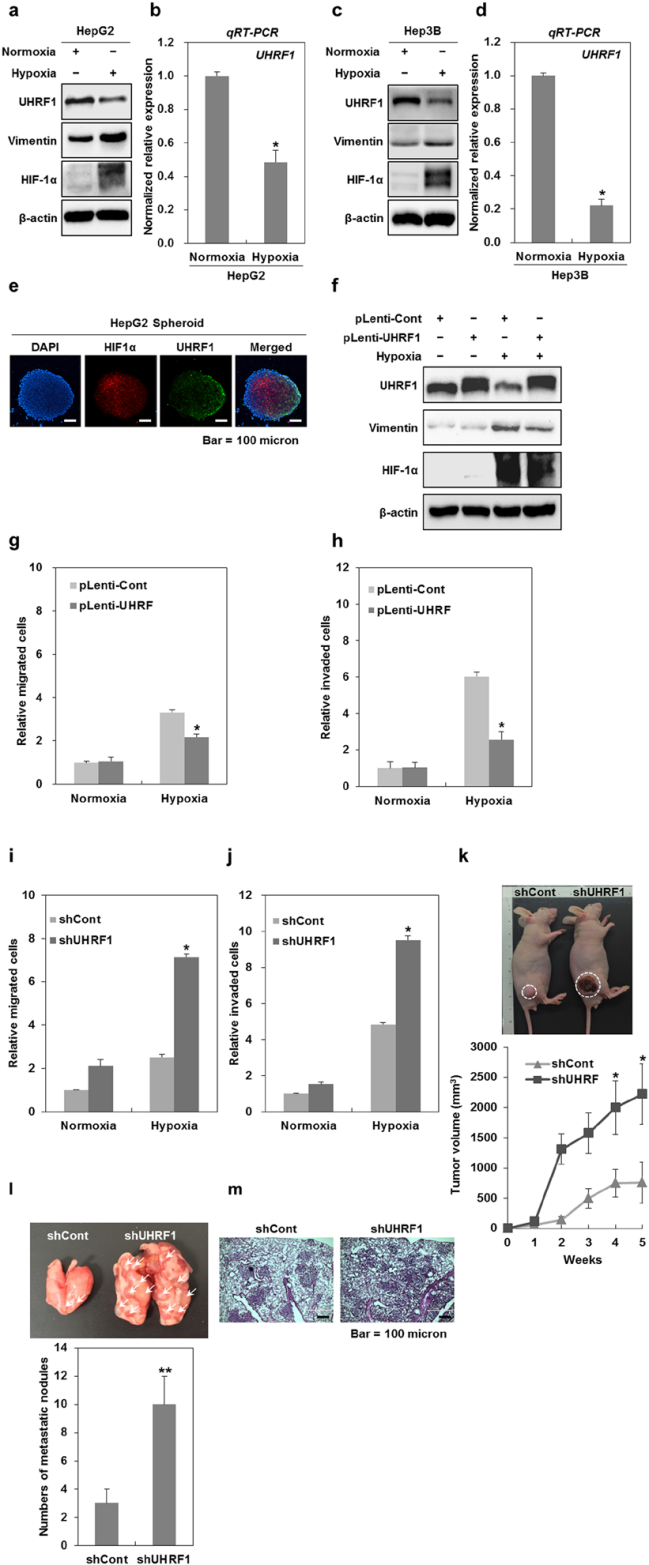
Hypoxia-induced downregulation of UHRF1 contributes to the occurrence of EMT. (**a**) Western blot analysis for UHRF1, vimentin and HIF-1α in HepG2 cells grown under normoxia and hypoxia for 24 h. (**b**) qRT-PCR analysis for the mRNA expression level of UHRF1 in HepG2 cells grown under normoxia and hypoxia for 24 h. (**c**) Western blot analysis for UHRF1, vimentin and HIF-1α in Hep3B cells grown under normoxia and hypoxia for 24 h. (**d**) qRT-PCR analysis for the mRNA expression level of UHRF1 in Hep3B cells grown under normoxia and hypoxia for 24 h. (**e**) Immunocytochemistry for HIF-1α and UHRF1 in a HepG2 spheroid. (**f**) Western blot analysis for UHRF1, vimentin and HIF-1α in UHRF1-overexpressing HepG2 cells. (**g** and **h**) Effects of UHRF1 overexpression on the migratory and invasive properties of HepG2 cells grown under hypoxia for 24 h. The migration and invasion assay were performed using the Transwell chamber. (**I** and **J**) Effects of UHRF1 deficiency on the migratory and invasive properties of HepG2 cells grown under hypoxia for 24 h. The migration and invasion assay were performed using the Transwell chamber. (**k**) Effects of UHRF1 deficiency on tumorigenic capacity of HepG2 cells *in vivo*. shCont- and shUHRF1-HepG2 cells were subcutaneously injected to the right flank of athymic BALB/c female nude mice (n = 5 per group). After 5 weeks, tumor mass was photographed under a light microscope (up). The average of tumor volumes was measured at 1-week intervals (down). (**l**) Quantification of lung metastasis determined by counting the number of foci on the lung surface after tail vein injection of shCont- and shUHRF1-HepG2 cells (n = 4 per group). (**m**) H&E staining of lung sections after tail vein injection of shCont- and shUHRF1-HepG2 cells. β-actin was used as a loading control. Results from three independent experiments are expressed as means ± SEMs. (*P < 0.05, **P < 0.01).

Hypoxia-induced EMT is known to promote both the migration and invasiveness of cancer cells^27, 28^. Therefore, we investigated whether hypoxia can affect UHRF1 expression in HepG2 cells. As shown in Fig. 2a and b, both the protein and mRNA levels of UHRF1 were decreased under hypoxia, whereas vimentin expression was markedly increased. Furthermore, we also obtained similar results in Hep3B cells (Fig. 2c and d, Supplementary Fig. S1g and h).

To examine the level of UHRF1 under endogenous hypoxic conditions that closely recapitulate the *in vivo* condition, we used a multicellular tumor spheroid model. This model shows a gradient of oxygen caused by a hypoxic core^29, 30^. As shown in Fig. 2e, our confocal microscopy observation revealed that UHRF1 expression was decreased in the cells in hypoxic regions that remained positive for HIF-1a but not in the cells of the outer layer of a HepG2 spheroid.

Next, we investigated whether UHRF1 downregulation contributes to hypoxia-induced EMT in HepG2 cells. As shown in Fig. 2f–h, UHRF1 overexpression attenuated the increase in vimentin induced by hypoxia and reduced hypoxia-induced migration and invasiveness, indicating that hypoxia-mediated downregulation of UHRF1 is involved in EMT induction.

Moreover, we assessed the effect of UHRF1 deficiency on hypoxia-induced migration and invasiveness in HepG2 cells. UHRF1 deficiency promoted enhanced migration and invasiveness under hypoxia, indicating that UHRF1 downregulation may be a key event in hypoxia-induced malignancy (Fig. 2i and j).

As UHRF1 downregulation increased both migration and invasion *in vitro* and is involved in hypoxia-induced EMT, we investigated whether it contributes to tumor growth *in vivo*. In this regard, UHRF1-deficient HepG2 cells and their parental cells were engrafted into mice, and tumor formation was assessed. As shown in Fig. 2k, the tumor mass was dramatically greater in mice transplanted with UHRF1-deficient HepG2 cells compared with those that received the parental cells.

To assess the metastatic ability of UHRF1-deficient HepG2 cells *in vivo*, UHRF1-deficient HepG2 cells and their parental cells were injected into the tail veins of mice, and their metastatic ability was evaluated. As shown in Fig. 2l, the lung metastatic ability of UHRF1-deficient HepG2 cells was higher than that of their parental cells. Histological analysis confirmed that the extent of metastatic lesions caused by UHRF1-deficient HepG2 was greatly increased in lung sections (Fig. 2m). These results suggest that UHRF1 downregulation may be a critical event for tumor malignancy *in vivo*.

### Epigenetic modulation caused by UHRF1 deficiency leads to CXCR4 upregulation

**Figure 3:**
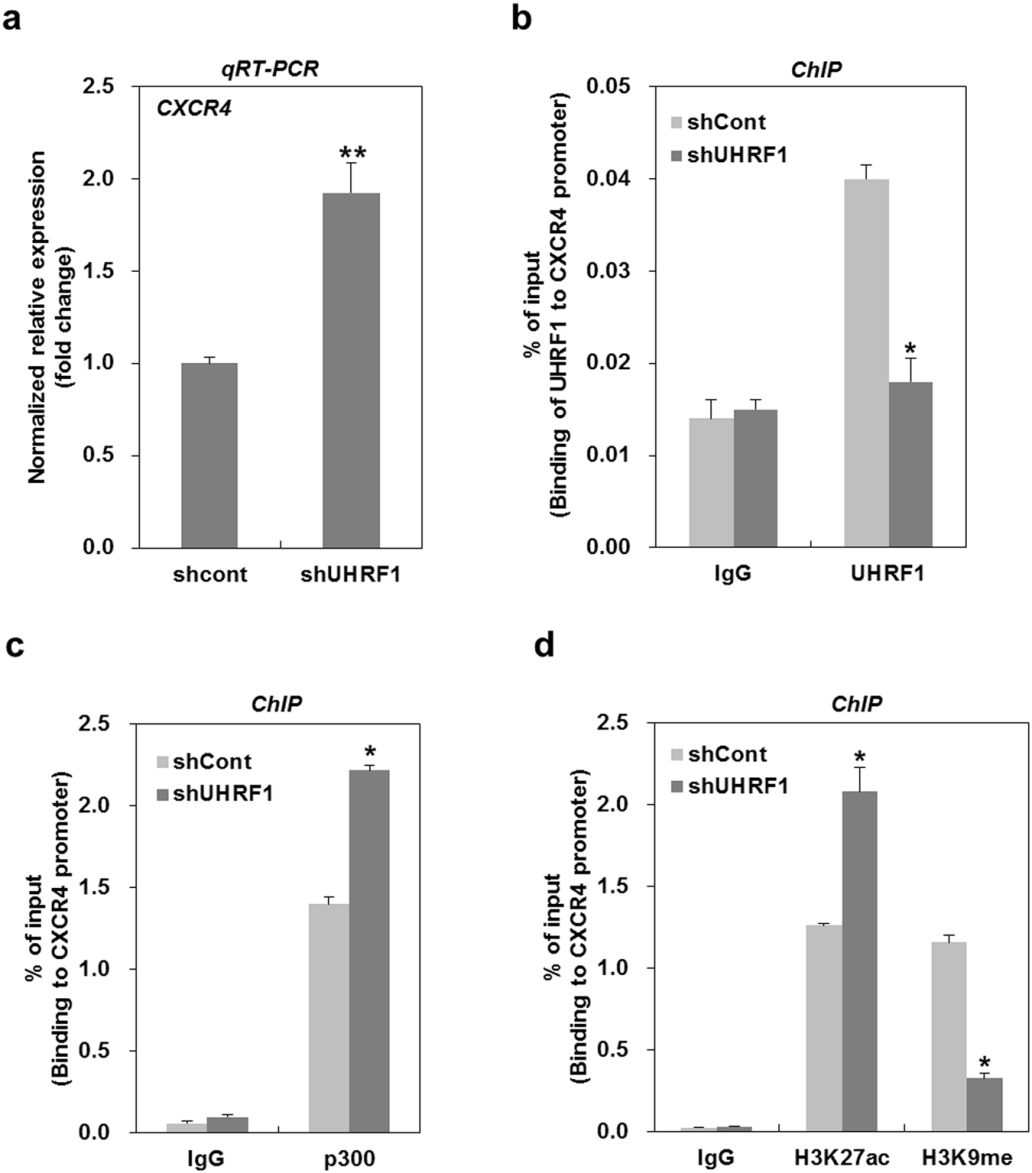
Downregulation of UHRF1 leads to upregulation of CXCR4 via epigenetic modulation. (**a**) qRT-PCR analysis for the mRNA expression level of CXCR4 in shCont- and shUHRF1-HepG2 cells. (**b**) ChIP analysis for the presence of UHRF1 on the CXCR4 promoter in shCont- or shUHRF1-HepG2 cells. Chromatin was immunoprecipitated with an anti-UHRF1 antibody. (**c**) ChIP analysis for the presence of p300 on the CXCR4 promoter in shCont- or shUHRF1-HepG2 cells. Chromatin was immunoprecipitated with an anti-p300 antibody. (**d**) ChIP analysis for the presence of H3K27ac and H3K9me on the CXCR4 promoter in shCont- or shUHRF1-HepG2 cells. Chromatin was immunoprecipitated with an anti-H3K27ac or -H3K9me antibody. The results show the percentage of the input chromatin (1%). IgG was used as the antibody control. Results from three independent experiments are expressed as means ± SEMs. (*P < 0.05, **P < 0.01).

We previously reported that UHRF1 deficiency upregulates CXCR4, thereby leading to EMT^25^. In this study, we confirmed an increase in the mRNA expression of CXCR4 in UHRF1-deficient HepG2 cells (Fig. 3a). As such, we further studied the molecular mechanism underlying this event. Because UHRF1 contributes to the methylation of CpG islands near the transcription start site (TSS) via recruiting DNMT1^1, 2^, we investigated whether UHRF1 deficiency influences DNA methylation of the CpG island near the CXCR4 gene promoter. Bisulfite sequencing analysis showed that the CpG island of the CXCR4 gene promoter was hypomethylated in both UHRF1-deficient HepG2 and their parental cells, indicating that an increase of CXCR4 by UHRF1 deficiency was not associated with the DNA methylation pattern in the CpG island of the CXCR4 gene promoter (Supplementary Fig. S2a).

UHRF1 has been reported to play a key role in transcriptional repression by directly binding to target promoters^31^. Thus, to determine whether UHRF1 modulates the mRNA expression of CXCR4 by binding to the CXCR4 promoter, we performed chromatin immunoprecipitation analysis using a primer located in a region upstream from the CXCR4 TSS (Supplementary Fig. S2b). UHRF1 occupied the promoter of the CXCR4 gene in the parental cells, and this occupancy was significantly decreased in UHRF1-deficient HepG2 cells (Fig. 3b). Conversely, the binding of p300, a transcriptional co-activator, to the promoter of CXCR4 in UHRF1-deficient HepG2 cells was increased compared with that in the parental cells (Fig. 3c). Moreover, the acetylation of lysine 27 on histone H3 (H3K27ac), an active mark for transcription, in the promoter was enhanced in UHRF1-deficient HepG2, whereas methylation of lysine 9 on histone H3 (H3K37me3), a repressive mark for transcription, was significantly decreased (Fig. 3d). Collectively, these results suggest that the increased level of CXCR4 caused by UHRF1 deficiency may be due either to the loss of its transcriptional repression function or changes in histone modification on the CXCR4 promoter.

### The secretion of IL-6 induced by UHRF1 deficiency leads to the induction of EMT

**Figure 4:**
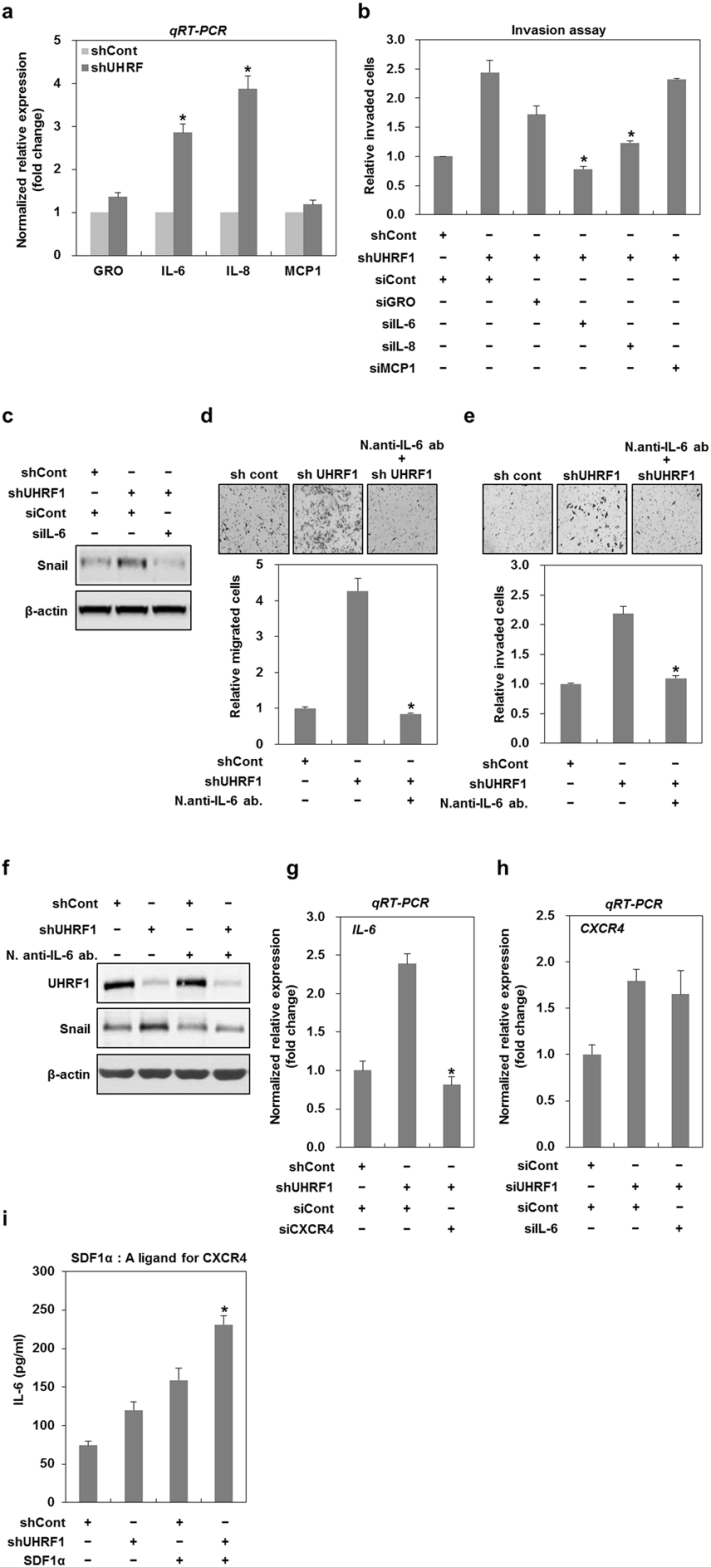
Downregulation of UHRF1 induces upregulation of IL-6 promoting EMT. (**a**) qRT-PCR analysis for the mRNA expression levels of GRO, IL-6, IL-8 and MCP1 in shCont- and shUHRF1-HepG2 cells. (**b**) Effects of siRNAs targeting GRO, IL-6, IL-8 and MCP1 on the invasive property of shUHRF1-HepG2 cells. The cells were grown in the presence or absence of siRNAs for 48 h, and the invasion assay was performed using the Transwell chamber. (**c**) Effect of siRNA targeting IL-6 on the protein expression level of Snail in shUHRF1-HepG2 cells. The cells were grown in the presence or absence of siRNA targeting IL-6 for 48 h, and western blot analysis was performed. (**d** and **e**) Effects of a neutralizing antibody of IL-6 on the migratory and invasive properties of shUHRF1-HepG2 cells. The cells were grown in the presence or absence of a neutralizing antibody of IL-6 for 48 h, and the migration and invasion assay were performed using the Transwell chamber. (**f**) Effects of a neutralizing antibody of IL-6 on the protein expression level of Snail in shUHRF1-HepG2 cells. The cells were grown in the presence or absence of a neutralizing antibody of IL-6 for 48 h, and western blot analysis was performed. (**g**) Effect of siRNA targeting CXCR4 on the mRNA expression of IL-6 in shUHRF1-HepG2 cells. The cells were grown in the presence or absence of siRNA targeting CXCR4 for 48 h, and qRT-PCR analysis was performed. (**h**) Effect of siRNA targeting IL-6 on the mRNA expression of CXCR4 in shUHRF1-HepG2 cells. The cells were grown in the presence or absence of siRNA targeting IL-6 for 48 h, and qRT-PCR analysis was performed. (**i**) Effect of SDF1α (250 ng/ml) on the secretion of IL-6 from shUHRF1-HepG2 cells. The cells were grown in the presence or absence of SDF1α for 48 h, and ELISA was performed to detect the secretion of IL-6 using conditioned medium obtain from the cells treated with SDF1α. β-actin was used as a loading control. Results from three independent experiments are expressed as means ± SEMs. (*P < 0.05, **P < 0.01).

Because many cytokines are involved in the induction of EMT^32, 33^, we sought to investigate which cytokines are secreted from UHRF1-deficient HepG2 cells. In this regard, we analyzed the amount of cytokines secreted by the cells into the cultured medium. Higher levels of GRO, IL-6, IL-8 and MCP1 were present in the culture medium from UHRF1-deficient HepG2 cells compared with their parental cells (Supplementary Fig. S3a). To further assess the level of these cytokines, we performed qRT-PCR analysis. The mRNA expression levels of IL-6 and IL-8 were markedly increased in UHRF1-deficient HepG2 cells, whereas the extent to which the mRNA expression of GRO or MCP1 increased was relatively low (Fig. 4a).

We next scrutinized the degree to which these individual cytokines contribute to the invasiveness of UHRF1-deficient HepG2 cells. To this end, cells were transfected with siRNAs targeting these cytokines (Supplementary Fig. S3b). IL-6 siRNA effectively suppressed the invasive property of UHRF1-deficient HepG2 cells, whereas GRO- or IL-8 siRNA partially reduced invasiveness, and MCP1 siRNA had no effect (Fig. 4b). In addition, IL-6 siRNA efficiently suppressed the increased expression of Snail, an EMT-regulating transcription factor, in UHRF1-deficient HepG2 cells (Fig. 4c). These results strongly suggest that IL-6 is a key contributor to the EMT phenotype observed in UHRF1-deficient HepG2 cells.

We further confirmed the role of IL-6 in EMT using a neutralizing antibody against IL-6. Treatment with this antibody effectively attenuated both the migratory and invasive properties of UHRF1-deficient HepG2 cells (Fig. 4d and e). Consistent with these findings, Snail was significantly reduced by treatment with this antibody (Fig. 4f).

To determine whether IL-6 is associated with CXCR4, we transfected UHRF1-deficient HepG2 cells with CXCR4- or IL-6 siRNA (Supplementary Fig. S3b and c). Interestingly, CXCR4 siRNA significantly attenuated the mRNA expression of IL-6, whereas IL-6 siRNA did not modulate that of CXCR4, suggesting that CXCR4 is an upstream activator of IL-6 (Fig. 4g and h). In addition, to determine the involvement of CXCR4 in IL-6 secretion, cells were treated with SDF1α, a specific ligand for CXCR4. The increased secretion of IL-6 was detected in UHRF1-deficient HepG2 cells treated with SDF1α (Fig. 4i). These results indicated that CXCR4 is necessary for IL-6 upregulation, thereby leading to EMT in UHRF1-deficient HepG2 cells.

### UHRF1 deficiency increases EMT via activating the IL-6/JAK/STAT3 signaling pathway

**Figure 5:**
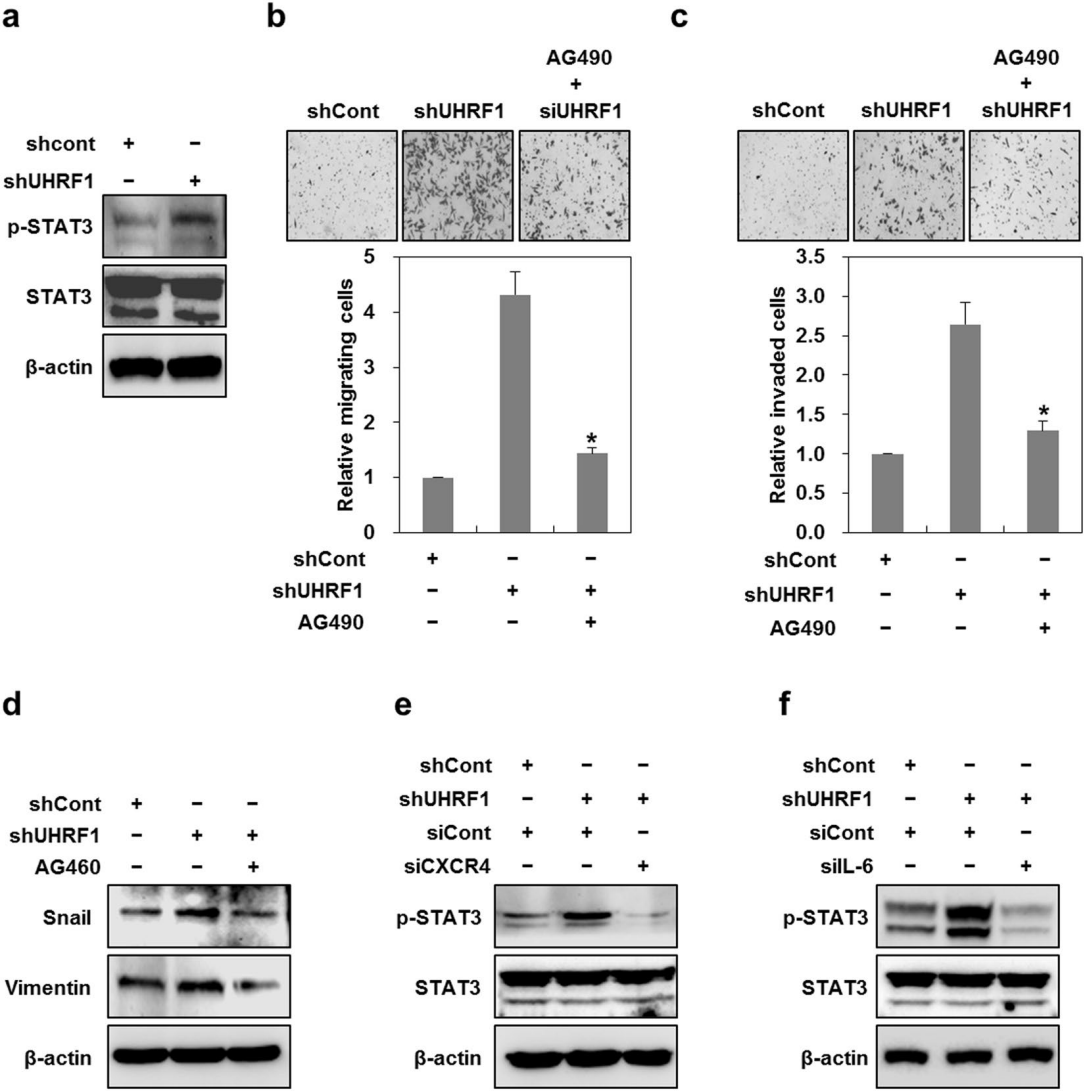
Downregulation of UHRF1 activates CXCR4/IL-6/JAK/STAT3 pathway. (**a**) Western blot analysis for the phosphorylation status of STAT3 in shCont- and shUHRF1-HepG2 cells. (**b** and **c**) Effects of AG490 on the migratory and invasive properties of shUHRF1-HepG2 cells. The cells were grown in the presence or absence of AG490 (5 μM) for 48 h, and the migration and invasion assay were performed using the Transwell chamber. (**d**) Effects of AG490 on the protein expression level of Snail and vimentin in shUHRF1-HepG2 cells. (**e**) Effects of siRNA targeting CXCR4 on the protein expression level of phosphorylated STAT3 in shUHRF1-HepG2 cells. The cells were grown in the presence or absence of siRNA targeting CXCR4 for 48 h, and western blot analysis was performed. (**f**) Effects of siRNA targeting IL-6 on the protein expression level of phosphorylated STAT3 in shUHRF1-HepG2 cells. The cells were grown in the presence or absence of siRNA targeting IL-6 for 48 h, and western blot analysis was performed. β-actin was used as a loading control. Results from three independent experiments are expressed as means ± SEMs. (*P < 0.05, **P < 0.01).

As the activation of the JAK/STAT pathway is a prime signaling event caused by various cytokines and growth factors^34^, we investigated whether IL-6 positively regulates EMT in UHRF1-deficient HepG2 cells by activating this pathway. As shown in Fig. 5a, phosphorylated STAT3 was increased in UHRF1-deficient HepG2 cells compared with their parental cells. We also obtained a similar result in UHRF1-deficient Hep3B cells (Supplementary Fig. S3d). To examine the potential involvement of JAK/STAT3 in EMT, we treated the UHRF1-deficient HepG2 cells with AG490, a JAK inhibitor. As expected, AG490 greatly suppressed the migratory and invasive properties of UHRF1-deficient HepG2 cells and efficiently attenuated the increase in Snail and vimentin (Fig. 5b–d). When we treated the cells with siRNA targeting CXCR4 or IL-6, these siRNAs significantly reduced the increased level of phosphorylated STAT3 in UHRF1-deficient HepG2 cells, indicating that JAK/STAT3 activation is a downstream event in the CXCR4/IL-6 signaling cascade (Fig. 5e and f). These results clearly show that activation of the CXCR4/IL-6/JAK/STAT3 pathway is required for EMT caused by UHRF1 deficiency.

### UHRF1 deficiency promotes EMT via the activation of AKT/JNK signaling

**Figure 6:**
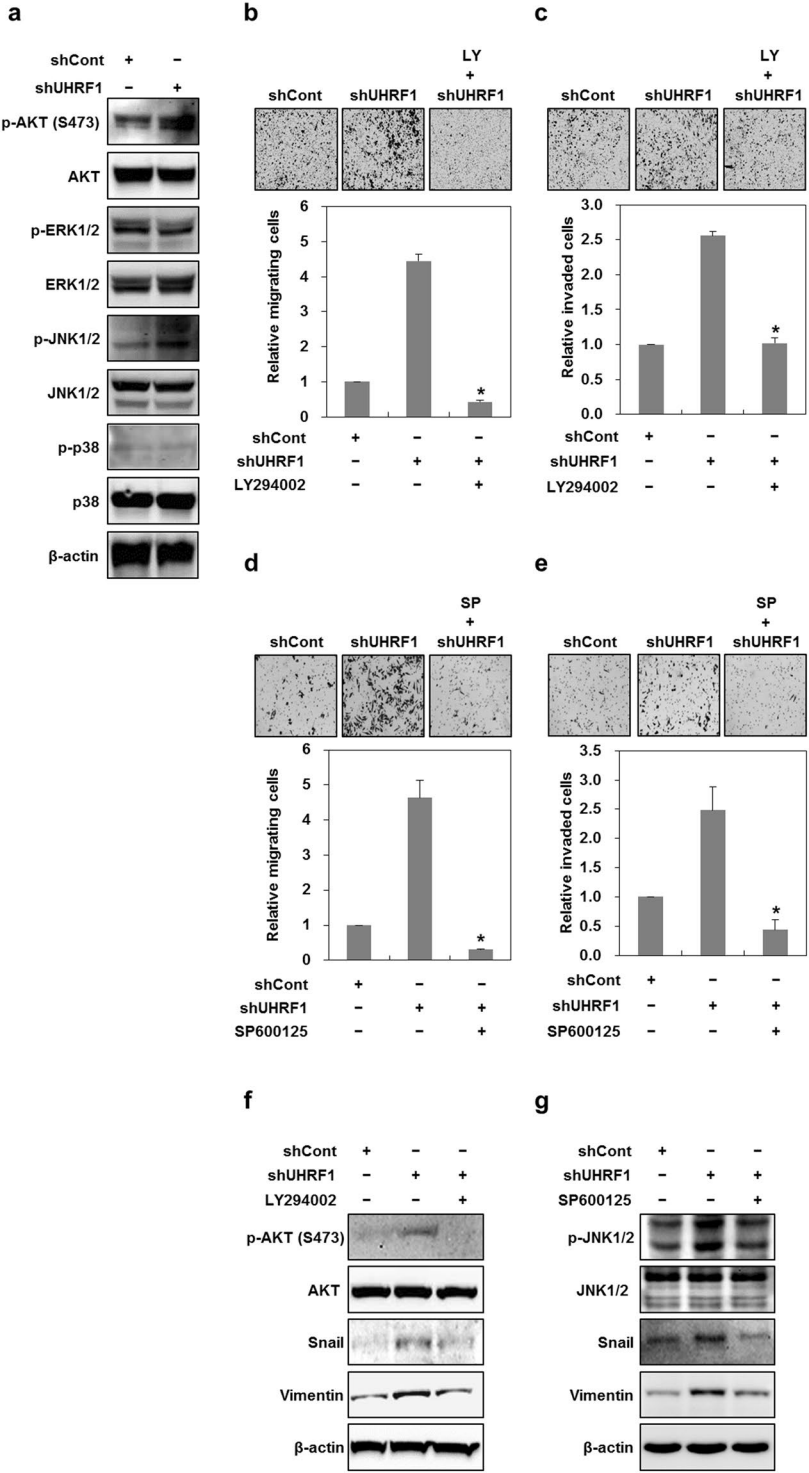
Downregulation of UHRF1 activates AKT and JNK. (**a**) Western blot analysis for the phosphorylation status of AKT and MAPKs in shCont- and shUHRF1-HepG2 cells. (**b** and **c**) Effects of LY294002 on the migratory and invasive properties of shUHRF1-HepG2 cells. The cells were grown in the presence or absence of LY294002 (2 μM) for 48 h, and the migration and invasion assay were performed using the Transwell chamber. (**d** and **e**) Effects of SP600125 on the migratory and invasive properties of shUHRF1-HepG2 cells. The cells were grown in the presence or absence of SP600125 (5 μM) for 48 h, and the migration and invasion assay were performed using the Transwell chamber. (**f**) Effects of LY294002 on the protein expression level of phosphorylated AKT, Snail and vimentin in shUHRF1-HepG2 cells. (**g**) Effects of SP600125 on the protein expression level of phosphorylated JNK, Snail and vimentin in shUHRF1-HepG2 cells. β-actin was used as a loading control. Results from three independent experiments are expressed as means ± SEMs. (*P < 0.05, **P < 0.01).

**Figure 7:**
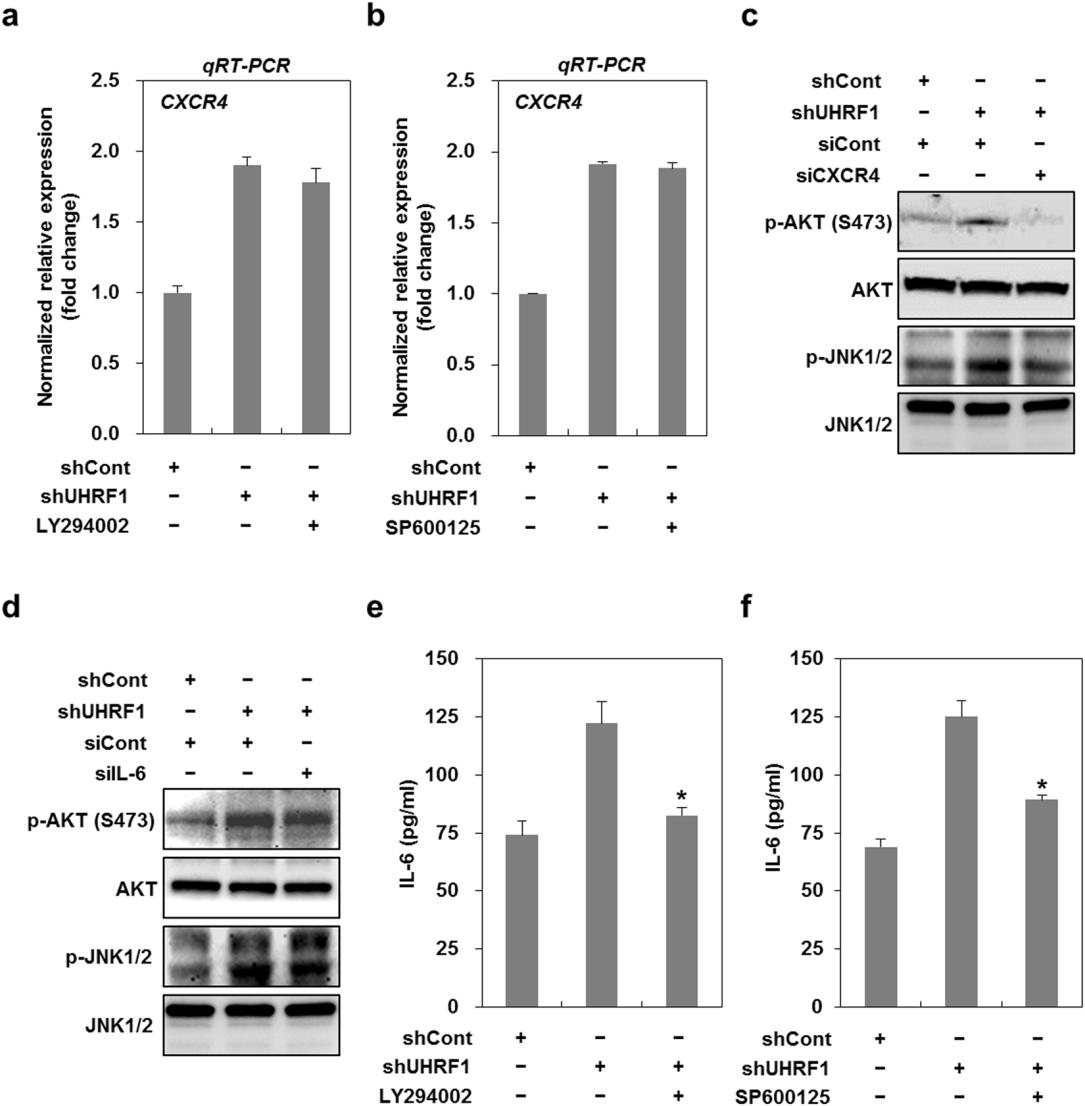
The activation of CXCR4/AKT-JNK/IL-6/JAK/STAT3 pathway is required for the induction of EMT caused by downregulation of UHRF1. (**a**) Effect of LY294002 on the mRNA expression of CXCR4 in shUHRF1-HepG2 cells. The cells were grown in the presence or absence of LY294002 (2 μM) for 48 h, and qRT-PCR analysis was performed. (**b**) Effect of SP600125 on the mRNA expression of CXCR4 in shUHRF1-HepG2 cells. The cells were grown in the presence or absence of SP600125 (5 μM) for 48 h, and qRT-PCR analysis was performed. (**c**) Effects of siRNA targeting CXCR4 on the protein expression level of phosphorylated AKT and -JNK in shUHRF1-HepG2 cells. The cells were grown in the presence or absence of siRNA targeting CXCR4 for 48 h, and western blot analysis was performed. (**d**) Effects of siRNA targeting IL-6 on the protein expression level of phosphorylated AKT and -JNK in shUHRF1-HepG2 cells. The cells were grown in the presence or absence of siRNA targeting IL-6 for 48 h, and western blot analysis was performed. (**e**) and (**f**) Effect of LY294002 or SP600125 on the secretion of IL-6 from shUHRF1-HepG2 cells. The cells were grown in the presence or absence of LY294002 (2 μM) or SP600125 (5 μM) for 48 h, and ELISA was performed to detect the secretion of IL-6 using conditioned medium obtain from the cells treated with LY294002 or SP600125. β-actin was used as a loading control. Results from three independent experiments are expressed as means ± SEMs. (*P < 0.05, **P < 0.01).

NICD1 (Notch Intracellular domain 1) and β-catenin have been associated with EMT activation^35, 36^. Furthermore, CXCR4 is known to activate the phosphoinositide 3-kinase (PI3K)/AKT or mitogen-activated protein kinase (MAPK) pathway^37^. Thus, we examined whether these signaling pathways contribute to EMT in UHRF1-deficient HepG2 cells. As shown in Supplementary Fig. S4a, UHRF1-deficient HepG2 cells showed a decreased level of NICD1 and a consistent level of β-catenin, indicating that NCID1 and β-catenin do not appear to be involved. However, these cells exhibited a significant increase in phosphorylated AKT and JNK (Fig. 6a). We also obtained similar results in HepG2 cells that had been transiently transfected with UHRF1 siRNA, and further confirmed them in Hep3B cells that had been stably transfected with UHRF1 shRNA (Supplementary Fig. S4b and c). To assess the involvement of AKT or JNK in EMT, we treated the cells with LY294002 or SP600125, inhibitors of the PI3K/AKT pathway and JNK, respectively. Both LY294002 and SP600125 greatly reduced migratory and invasive properties and effectively attenuated the increased level of Snail and vimentin in these cells (Fig. 6b–g). To elucidate the relationship among CXCR4, IL-6, AKT and JNK, UHRF1-deficient HepG2 cells were treated with siRNAs or inhibitors specific for these factors. Although treatment with LY294002 or SP600125 had no effect on the mRNA expression of CXCR4, treatment with siRNA targeting CXCR4 completely attenuated the increase in the phosphorylated forms of AKT and JNK, suggesting that CXCR4 is an upstream activator of AKT and JNK (Fig. 7a–c).

Moreover, IL-6 siRNA was ineffective in attenuating phosphorylated AKT and JNK, whereas LY294002 or SP600125 efficiently blocked both the mRNA expression and secretion of IL-6 and also attenuated the increased level of phosphorylated STAT3 (Fig. 7d–f and Supplementary Fig. S4d–g). This result indicates that IL-6 is a downstream target of AKT and JNK.

To further identify whether there was any cross-talk between AKT and JNK, we assessed the changes in phosphorylated AKT or JNK after treatment with LY294002 and SP600125. LY294002 effectively attenuated the increased level of phosphorylated JNK, and SP600125 also greatly modulated that of phosphorylated AKT, indicating that there is a positive feedback regulation between AKT and JNK (Supplementary Fig. S4h and i).

Taken together, these results strongly suggest that the activation of the CXCR4/AKT-JNK/IL-6 pathway is required for UHRF1 deficiency-induced EMT.

### UHRF1 deficiency leads to the expansion of cancer stem-like cells

**Figure 8:**
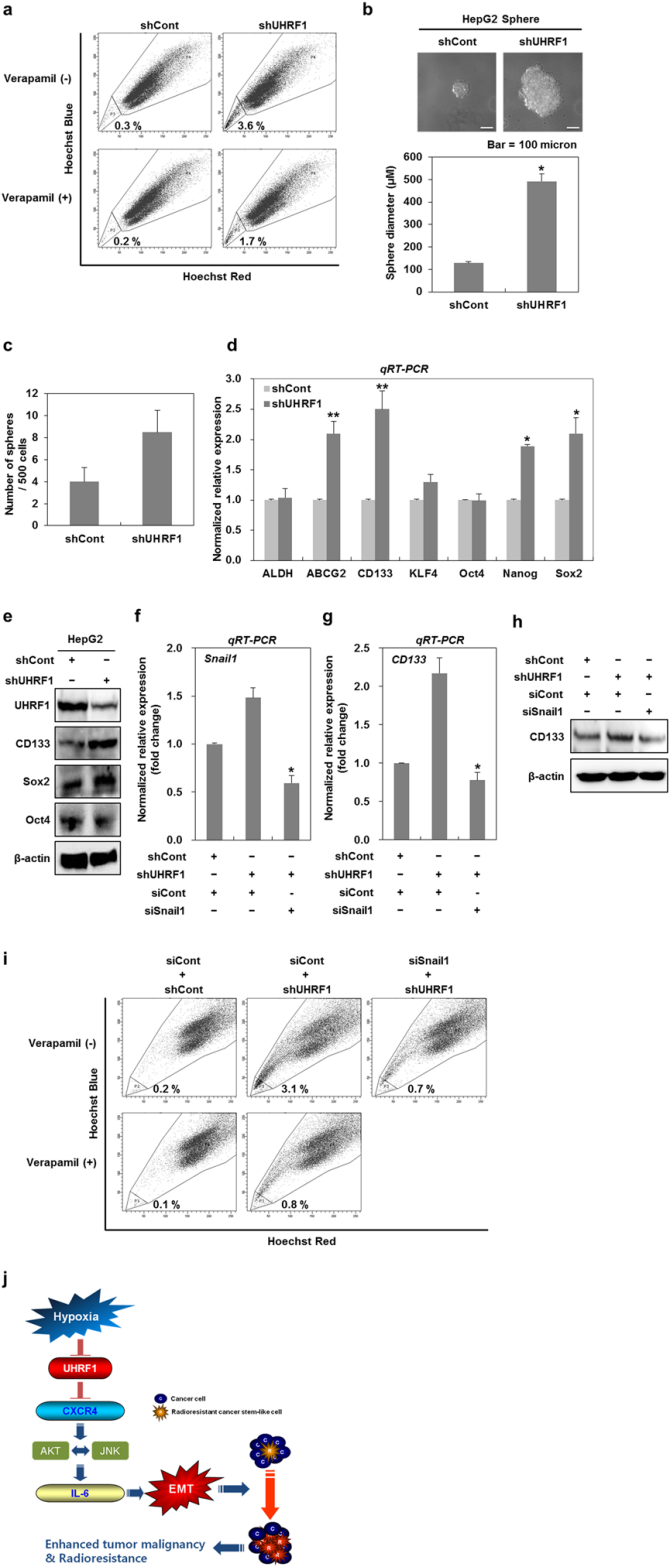
Downregulation of UHRF1 contributes to the expansion of cancer stem-like cells. (**a**) Flow cytometry analysis for the fraction of SP in shCont- and shUHRF1-HepG2 cells. shCont- and shUHRF1-HepG2 cells were stained with 5 μg/ml of Hoechst 33342 in the presence or absence of verapamil (50 μM), and the fraction of SP in the cells was analyzed using fluorescence-activated cell sorting. (**b** and **c**) Quantification of sphere-forming abilities of shCont- and shUHRF1-HepG2 cells. The cells were grown in DMEM/F12 supplemented with B27, N2, basic fibroblast- and epidermal growth factor onto 24-well ultra low attachment plates at 500 cells per well for 7 days, and the size and number of spheres were determined. To measure the size of sphere, 12 spheres per group were randomly selected. (**d**) qRT-PCR analysis for the mRNA expression levels of cancer stem cell-related factors in shCont- and shUHRF1-HepG2 cells. (**e**) Western blot analysis for CD133, Sox2 and Oct4 in shCont- and shUHRF1-HepG2 cells. (**f** and **g**) Effect of siRNA targeting Snail1 on the mRNA expression of CD133 in shUHRF1-HepG2 cells. The cells were grown in the presence or absence of siRNA targeting Snail1 for 48 h, and qRT-PCR analysis was performed. (**h**) Effect of siRNA targeting Snail1 on the protein expression level of CD133 in shUHRF1-HepG2 cells. The cells were grown in the presence or absence of siRNA targeting Snail1 for 48 h, and western blot analysis was performed. (**i**) Effect of siRNA targeting Snail1 on the fraction of SP in shURHF1 HepG2 cells. The cells were grown in the presence or absence of siRNA targeting Snail1 for 48 h, and the cells were stained with 5 μg/ml of Hoechst 33342 in the presence or absence of verapamil (50 μM). The fraction of SP in the cells was analyzed using fluorescence-activated cell sorting. (**j**) Schematic model underlying UHRF1 deficiency-induced EMT in hepatocellular carcinoma cells. β-actin was used as a loading control. Results from three independent experiments are expressed as means ± SEMs. (*P < 0.05, **P < 0.01).

EMT is closely related to an increase in the cancer stem-like cell population^15, 38^. Therefore, we investigated whether UHRF1 deficiency also triggers changes in the numbers of cancer stem-like cells. As the fraction of side population (SP) cells shows characteristics representative of cancer stem-like cells^39^, we treated UHRF1-deficient HepG2 cells with Hoechst 33342. Flow cytometry analysis revealed that the fraction of SP in UHRF1-deficient HepG2 cells was approximately 3 times higher than that in their parental cells (Fig. 8a). Moreover, UHRF1-deficient HepG2 cells formed larger and more numerous spheres, indicating that these cells had higher sphere-forming ability (Fig. 8b and c). We also obtained a similar result in UHRF1-deficient Hep3B cells (Supplementary Fig. S5a).

Next, we determined the level of cancer stem-like cell-related factors in UHRF1-deficient HepG2 cells. These cells showed an increase in the mRNA levels of ABCG2, CD133, KLF4, Nanog and Sox2, but exhibited no significant change in the level of ALDH and Oct4 (Fig. 8d). These factors are known to induce cancer stem-like cells or stemness^15^. Western blot analysis also showed an increase in the protein levels of CD133 and Sox2 in UHRF1-deficient HepG2 cells (Fig. 8e). We further confirmed that the protein level of CD133 was also increased in UHRF1-deficient Hep3B cells (Supplementary Fig. S5b).

To determine whether UHRF1 deficiency-induced EMT affects the expansion of cancer stem-like cells, we transfected UHRF1-deficient HepG2 cells with Snail siRNA and assessed the mRNA and protein levels of CD133. Snail siRNA markedly attenuated the upregulation of CD133 at both the mRNA and protein levels (Fig. 8f–h). Moreover, Snail siRNA significantly suppressed an increase in both SP fraction and the size of spheres formed (Fig. 8i and Supplementary Fig. S5c).

In addition, both SP600125 and LY294002 effectively suppressed sphere formation in UHRF1-deficient HepG2 cells (Supplementary Fig. S5d and e). These data collectively show that UHRF1 deficiency-induced activation of AKT or JNK also contributes to the expansion of cancer stem-like cells.

## Discussion

Tumor malignancy is characterized by invasiveness and metastasis, which occur through EMT^8, 9^. Although EMT-associated epigenetic pathways have been widely explored^40^, the epigenetic mechanisms underlying the acquisition of malignancy are not completely understood. We recently showed that UHRF1 deficiency contributes to the acquisition of tumor malignancy in a diverse range of cancer cells^25^. In this current study, we further investigated the role of UHRF1 deficiency in human hepatocellular carcinoma cells. We found that the cells possessing low levels of UHRF1 had a high ability for migration and invasion compared with cells possessing abundant UHRF1. In parallel with these observations, the negative modulation of UHRF1 in cells possessing abundant UHRF1 increased the migratory and invasive properties of these cells, as well as tumor growth and lung metastasis. These observations strongly indicate that UHRF1 deficiency is closely associated with the malignant transformation of hepatocellular carcinoma cells.

Tumor suppressors including KiSS1, BRCA1, p16, MEG3 and PPAR-gamma are known to inhibit the tumor cell migration and invasion^41–45^. In addition, UHRF1 upregulation has been reported to increase the tumor cell migration and invasion via repressing these tumor suppressors^46^. On the basis of these previous reports, it can be easily expected that the UHRF1 downregulation can effectively represses the tumor cell migration and invasion. Contrary to this expectation, however, our observation revealed that UHRF1 downregulation greatly leads to increasing the migratory and invasive properties of cancer cells rather than decreasing them. As UHRF1 is an epigenetic regulator which can modulate gene expressions, the expressions of a variety of genes and their related signaling pathways can be changed by either upregulation or downregulation of UHRF1. Thus, our observation is thought to be due to the signaling pathways activated by UHRF1 deficiency. However, further studies are warranted to define the differences between upregulation and downregulation of UHRF1 in increasing migratory and invasive properties of cancer cells. UHRF1 upregulation was recently suggested to play a crucial role in the occurrence of primary hepatocellular carcinoma by acting as an oncogene^47, 48^. Furthermore, this upregulation can promote cellular proliferation by reducing either the transcriptional or protein level of tumor suppressors regulating cell growth^41, 49, 50^. Collectively, it is reasonable to assume that UHRF1 upregulation is closely related with the development, growth and maintenance of the primary tumor.

Hypoxic conditions are known to be a major trigger for EMT, which is involved in tumor malignancy^51, 52^. In this study, we first found that hypoxia downregulated UHRF1, but induced a mesenchymal marker. Interestingly, the forced expression of UHRF1 efficiently attenuated the increased migratory and invasive properties of HepG2 cells under hypoxia. Furthermore, because UHRF1 deficiency conferred HepG2 cells enhanced migratory and invasive abilities under hypoxia, UHRF1 appears to contribute to the suppression of hypoxia-induced EMT. This process is thought to occur via the UHRF1-mediated epigenetic repression of some of HIF1α- or EMT target genes. These results indicate that the hypoxia-mediated decrease in UHRF1 may play a crucial role in tumor malignancy, but additional studies examining its potential role in hypoxia-induced malignancy are warranted.

CXCR4 upregulation has been strongly implicated in tumor malignancy^19, 20, 53^. Consistent with these reports, we previously found that UHRF1 deficiency increased the level of CXCR4, thereby inducing EMT^25^. In this study, we further investigated the regulatory mechanism underlying the UHRF1 deficiency-induced upregulation of CXCR4. UHRF1 has been shown to suppress the activity of the p21 promoter by cooperating with G9a, a histone methyltransferase^31^. Similarly, we found that UHRF1 also directly binds to the CXCR4 promoter and appears to repress CXCR4 gene expression. The occupancies of UHRF1 and H3K27me3 on the CXCR4 promoter were significantly decreased by UHRF1 deficiency, whereas the occupancies of p300 and H3K27ac were both increased. UHRF1 can bind to G9a and HDAC1^31, 54^, and both G9a and HDAC1 repress transcription by modifying histones and chromatin structures^55^. Thus, the UHRF1 deficiency-mediated reduction and increase in the occupancy of H3K9me and H3K27ac are thought to be due to the decreased recruitment of G9a and HDAC1, respectively, on histone H3. Collectively, these results suggest a possible function of UHRF1 in directly modulating CXCR4 expression by interacting with its promoter region.

Inflammatory cytokines promotes tumor malignancy by leading to EMT^32, 56^. In particular, IL-6 is a potent trigger for EMT in many cancer types^56, 57^. Accordingly, we found that the UHRF1 deficiency-induced upregulation of CXCR4 promoted IL-6 expression and secretion. Consequently, the migratory and invasive properties of HepG2 cells were increased through the IL-6/JAK/STAT3/Snail signaling axis. These results are supported by several reports suggesting that IL-6 is a downstream target of the CXCR4 signaling pathways^58, 59^, and tumor malignancy occurs through the IL-6-induced activation of the JAK/STAT3/Snail pathway^60^.

Importantly, CXCR4 activates PI3K/AKT or MAPKs in various cancer cell types^37^. Consistent with these findings, we found that UHRF1 deficiency-induced upregulation of CXCR4 also activated AKT and JNK, thereby subsequently inducing the expression of IL-6. Interestingly, AKT and JNK positively regulate each other’s activity and closely cooperate in inducing EMT. Although the regulatory mechanism underlying the positive feedback activation between AKT and JNK remains unclear, the following notions may support these results. The PI3K/AKT or the PI3K/Rac1 signaling pathway activates JNK and promotes cell proliferation and EMT^61, 62^. Additionally, JNK acts as an upstream activator of AKT in cell survival^63^.

In this study, UHRF1 deficiency led to the expansion of the population of cancer stem-like cells. Additionally, the expression of cancer stem cells-related genes was increased by UHRF1 deficiency. EMT has been reported to contribute to an increase in the population of cancer stem-like cells^15, 38^. Consistent with these reports, the transient knockdown of Snail efficiently modulated not only the expression of CD133 but also the expansion of the population of cancer stem-like cells caused by UHRF1 deficiency. The activation of the AKT-JNK/Snail pathway by UHRF1 deficiency was required for the expansion of these cells. Interestingly, UHRF1 levels were decreased in sphere-forming HepG2 cells compared with monolayer HepG2 cells (Supplementary Fig. S6a). In addition, UHRF1 was downregulated to a greater extent in CD133^+^ cells compared with CD133^−^ cells, indicating that UHRF1 deficiency may contribute to the maintenance of cancer stem-like cells (Supplementary Fig. S6b and c).

In conclusion, UHRF1 deficiency is closely correlated with tumor malignancy. Furthermore, we present evidence that UHRF1 deficiency leads to the induction of EMT by activating the CXCR4/AKT-JNK/IL-6 signaling pathway, thereby contributing to the expansion of cancer stem-like cells (Fig. 8j).

## Materials and Methods

### Reagents

All antibodies used in this study are listed in Supplementary Table S7. The chromatin Immunoprecipitation Kit and SP600125 were purchased from Millipore (Darmstadt, Germany). DAPI (4,6-diamidino- 2-phenylindole) and Verapamil were purchased from Sigma-Aldrich (St Louis, MO, USA). Recombinant SDF1α (CXCL12) and human Cytokine Array Kit, Panel A, were purchased from R&D systems, Inc. (Minneapolis, MN, USA).

### Cell culture

The human hepatocellular carcinoma HepG2, Hep3B and Huh7 cells were obtained from the American Type Culture Collection (Manassas, VA, USA). Human normal fetal hepatocytes were obtained from Sciencell (Carlsbad, CA, USA). The detailed methodology is described in the Supplementary Methods.

### Establishment of knockdown or overexpression cell lines

shRNA constructs were purchased from Sigma-Aldrich. Overexpression constructs for human UHRF1 were purchased from ORIGENE Technologies, Inc. (Rockville, MD, USA). The detailed methodology is described in the Supplementary Methods.

### Small interfering RNA transfection

RNA interference mediated by siRNAs was achieved using double-stranded RNA molecules. The detailed methodology is described in the Supplementary Methods.

### *In vivo* tumor growth and Tail vein injection

All animal protocols used in this study were approved by the Institutional Animal Care and Use Committee at Dongnam Institute of Radiological & Medical Sciences (DIRAMS; Busan, Republic of Korea). All of the experimental procedures in this study were performed in accordance with the guidelines and regulations approved by Dongnam Institute of Radiological & Medical Sciences (DIRAMS; Busan, Republic of Korea). The detailed methodology is described in the Supplementary Methods.

### Immunohistochemistry

Athymic BALB/C nude mice were killed and tumor tissues were harvested and fixed in 4% formaldehyde, followed by cryoprotection with 30% sucrose for 24 h at 4 °C. The detailed methodology is described in the Supplementary Methods.

### Western blot analysis

Western blot analysis was performed as previously described^25^. Proteins were visualized using an enhanced chemiluminescence system (Amersham Biosciences, Piscataway, NJ, USA).

### Quantitative real-time PCR (qRT-PCR)

qRT-PCR was performed as previously described^25^. The primers used in this study are described in Supplementary Table S8. *GAPDH* was used as a control to normalize the expression value of the genes of interest. The relative expression level of the genes was calculated using the 2^−ΔΔCt^ method^64^.

### Migration and invasion assays

Cell migration and invasion assay were performed using the Transwell chamber. The detailed methodology is described in the Supplementary Methods.

### Tumor spheroid formation and immunocytochemistry

To generate tumor spheroids under non-adherent conditions, the cells were seeded in 24-well ultra-low-attachment plates (Corning Costar Corp., Cambridge, MA, USA). Immediately after harvesting, the spheroids were fixed and then embedded in OCT compound (Scigen Scientific Inc., Gardena, CA, USA). The detailed methodology is described in the Supplementary Methods.

### Assessments of IL-6 generation

Subconfluent cells (approximately 70% confluence) were washed, serum-free ECM was added, and the levels of IL-6 in the conditioned medium were measured using commercially available ELISA kits purchased from R&D Systems (Minneapolis, MN, USA).

### DNA methylation analysis via bisulfite sequencing (BSP)

Genomic DNA was extracted from cells using TRIZOL (Gibco), and then subjected to sodium bisulfite conversion using the EZ DNA Methylation-Gold Kit (Zymo Research Corp., Orange, CA, USA) according to the manufacturer’s instructions. The detailed methodology is described in the Supplementary Methods.

### Chromatin immunoprecipitation (ChIP)

ChIP assays were performed using a Magna ChIP kit (Millipore, Billerica, MA, USA) according to the manufacturer’s protocol. The primer used for this study is described in Supplementary Table S8. Anti-UHRF1, -p300, -H3K27ac and -H3K9me antibodies were used to immunoprecipitate the chromatin fragments.

### Sphere forming assay

The size and number of spheres were determined using a phase-contrast Nikon microscope (TS100; Tokyo, Japan). The detailed methodology is described in the Supplementary Methods.

### Fluorescence-activated cell sorting

The labeled cells were sorted by flow cytometry, FACSAria^TM^ (Special order system, BD Biosciences) into CD133^+^ and CD133^−^ cells. The detailed methodology is described in the Supplementary Methods.

### Statistical analyses

All data presented are representative of at least three independent experiments. Differences between groups were analyzed using Student’s t-test and one-way analysis of variance (ANOVA) or two-way ANOVA. P-values < 0.05 (indicated by * on figures) were considered significant.

## Electronic supplementary material

Supplementary information
