# Supplementary material for: Downregulation of UHRF1 increases tumor malignancy by activating the CXCR4/AKT-JNK/IL-6/Snail signaling axis in hepatocellular carcinoma cells
Source: Sci Rep. 2017 Jun 5;7:2798. doi: 10.1038/s41598-017-02935-2 (PMC5459852; doi:10.1038/s41598-017-02935-2)
Supplement: Supplementary file 1 — Supplementary information [file 41598_2017_2935_MOESM1_ESM.pdf]

Supplementary Information

Downregulation of UHRF1 increases tumor malignancy by activating the CXCR4/AKT-JNK/IL-6/Snail signaling axis in hepatocellular carcinoma cells

Ji-Hyun Kim[1], Jae-Woong Shim[1], Da-Young Eum[1], Sung Dae Kim[1], Si Ho Choi[1], Kwangmo Yang[1], Kyu Heo[1,*] and Moon-Taek Park[1,*]

[1] Research Center, Dongnam Institute of Radiological & Medical Sciences (DIRAMS), Busan 46033, Republic of Korea

Supplementary Methods

Cell culture

The human hepatocellular carcinoma HepG2, Hep3B and Huh7 cells were obtained from the American Type Culture Collection (Manassas, VA, USA). The cells were cultured in a humidified 5% CO_2 atmosphere at 37°C. HepG2 and Hep3B were maintained in Dulbecco's modified Eagle's medium supplemented with 10% (v/v) bovine calf serum, penicillin (50 units/mL), and streptomycin (50 μg/mL) (all from Gibco Invitrogen Corp., Carlsbad, CA, USA). Huh7 cells were maintained in RPMI 1640 (Welgene Inc., Daegu, Korea) supplemented with 10% (v/v) bovine calf serum, penicillin, and streptomycin. Human normal fetal hepatocytes (Sciencell, Carlsbad, CA, USA) were maintained in Hepatocyte Medium™ supplemented with 0.5% FBS, 0.1% hepatocyte growth supplement and 0.1% penicillin/streptomycin solution (all from Sciencell, Carlsbad, CA, USA). To examine the cellular response to hypoxia *in vitro*, cells were cultured for 6 h in a hypoxic chamber (Thermo Scientific., Waltham, MA, USA) containing 1% O_2, 5% CO_2 and 94% N_2 at 37°C.

Establishment of knockdown or overexpression cell lines

The appropriate double-stranded oligonucleotides [shUHRF1#1 (Cat# TRCN0000273315), shUHRF1#2 (Cat# TRCN0000004352), and its complement] were cloned into the pLKO.1-puro-shRNA vector (Sigma-Aldrich). For lentiviral particle production, 293 T cells were cultured in DMEM supplemented with 10% fetal calf serum, 2 mmol/L GlutaMAX (Gibco Invitrogen Corp.) and transfected with pLKO.1-puro-shRNA using the Lipofectamine 3000 reagent (Gibco Invitrogen Corp.) 48 hours after the transfection, viral supernatant was collected and passed through a 0.45-μm filter (Sartorius stedim biotech., Concord, CA, USA), and the viral supernatant was frozen at -80 °C. The supernatant was then used for transduction with supplementation of 4μg/mL Polybrene (Millipore). Subsequently, HepG2 cells were infected with viral particles, and shRNA-containing stable clones were selected using 0.5 μg/mL puromycin (Gibco Invitrogen Corp.). Endogenous UHRF1 knockdown was determined by western blot analysis using an anti-UHRF1 antibody.

Production of lentiviral particle containing pLenti-UHRF1-C-myc-DDK or its control vector was processed as described above for UHRF1 shRNA-containing cell lines. Subsequently, HepG2 cells were transiently infected with these viral particles. Overexpression of UHRF1 was determined by western blot analysis using an anti-UHRF1 or -FLAG (DDK) antibody.

Migration and invasion assays

The cell migration and invasion assay were performed using the Transwell chamber (8-μm pore size; BD Biosciences, San Jose, CA, USA). In total, 2×10^4 cells were resuspended in serum-free growth medium for both the cell migration and invasion assay. For the invasion assay, the interior of the inserts was precoated with 10 mg/mL growth factor-reduced Matrigel (BD Biosciences). For both assays, the cells were added to the interior of the inserts. Growth medium supplemented with 10% (v/v) bovine calf serum was added to the lower chamber. After incubation for 24 h, the cells attached on the upper surface of the filter were removed with a cotton swab. The cells on the lower surface of the filter were fixed and stained. The number of cells was determined by counting cells in five microscopic fields per well. Additionally, the cells were imaged by phase contrast microscopy (Nikon Eclipse 80i; Nikon, Tokyo, Japan).

Tumor spheroid formation and immunocytochemistry

To generate tumor spheroids under non-adherent conditions, the cells were seeded in 24-well ultra-low-attachment plates (Corning Costar Corp., Cambridge, MA, USA) at 3000 cells per well and cultured for 5 days. Immediately after harvesting, the spheroids were fixed and then embedded in OCT compound (Scigen Scientific Inc., Gardena, CA, USA). Afterward, the spheroids that were embedded in OCT compound were sectioned using a Cryotome FSE cryostat (Thermo Scientific) into 10 μm thick in accordance with the manufacturer's instructions, and stained overnight at 4°C with anti-UHRF1 (1:500) and -HIF1α (1:500) antibodies. After washing with PBS, 1:200 dilution of TEXAS RED-conjugated goat anti-rabbit IgG or anti-mouse IgG antibody in blocking solution was applied to the cryosections, and the samples were incubated for 60 min. The nuclei were counterstained with DAPI. The stained paraffin sections were observed and imaged using a confocal microscope (Carl Zeiss, Thornwood, NY, USA).

Small interfering RNA transfection

RNA interference mediated by siRNAs was achieved using double-stranded RNA molecules. The siRNAs against UHRF1 (sc-76805), Snail1 (sc-38398) or CXCR4 (sc-35421), IL-6 (sc-39627) were purchased from Santa Cruz Biotechnology, Incorporation. Control siRNA-A (sc-37007) (Santa Cruz) was used as a control. the siRNAs against GRO (5′-GGUAUGAUUAACUCUACCU-3′), IL-8 (5′-CUGUGUGUAAACAUGACUU-3′), MCP1 (5′-CUCCGAAGACUUGAACACU-3′) and the unrelated control siRNA (SN-1003) were purchased from Bioneer Corporation (Daejeon, Korea). Cells were grown to 30% confluency on 60-mm dishes and transfected with the siRNA duplexes (100 nM), using Lipofectamine 2000 (Gibco) in accordance with the manufacturer's instructions. Assays were performed 48 h after transfection.

***In vivo* tumor growth**

All animal protocols used in this study were approved by the Institutional Animal Care and Use Committee at Dongnam Institute of Radiological & Medical Sciences (DIRAMS; Busan, Republic of Korea). Female BALB/c nude (6-weeks old) were purchased from Japan SLC, Inc.(Shizuoka, Japan). Total number of 16 nude mice were randomly divided into two groups (shCont and shUHRF1; n=8 for each group) and each cell line (1×10^7) was inoculated subcutaneously into nude mice. Tumor volume was estimated as follows: tumor volume = $(\text{short axis})^2 \times (\text{long axis}) \times 0.5$. All animal studies were followed by a blind randomized animal study protocol.

Tail vein injection

The cells (1×10^6) were injected into tail veins of athymic BALB/C nude mice and allowed tumor formation for 2 months. When tumor was formed in the lung, the lung tissues were sectioned for immunohistochemistry. This study was reviewed and approved by the Institutional Animal Care and Use Committee at Dongnam Institute of Radiological & Medical Sciences (DIRAMS; Busan, Republic of Korea).

Immunohistochemistry

Athymic BALB/C nude mice were killed and tumor tissues were harvested and fixed in 4% formaldehyde, followed by cryoprotection with 30% sucrose for 72 h at 4°C. Afterward, the tissues were cut using a Cryotome FSE cryostat (Thermo Scientific) into 10 μm thick sections in accordance with the manufacturer's instructions. Sections were stained with H&E (hematoxylin and eosin). Additionally, the cells were imaged by phase contrast microscopy (Nikon Eclipse 80i; Nikon, Tokyo, Japan).

DNA methylation analysis via bisulfite sequencing (BSP)

Genomic DNA was extracted from cells using TRIZOL (Gibco), and then subjected to sodium bisulfite conversion using the EZ DNA Methylation-Gold Kit (Zymo Research Corp., Orange, CA, USA) according to the manufacturer's instructions. The bisulfite-converted genomic DNA was used for the methylation analysis of the CpG island of CXCR4 promoter (-1326 bp ~ -986 bp) with the PCR primer for DNA methylation. The primer for BSP was designed by using the MethPrimer program (http://www.urogene.org/methprimer/). The primer was as follows: forward (5′-TATTAGGGAGGGGTTTTAGATAAAG-3′) and reverse (5′-CCAAAAATAAACAAAAATTCCAAAC-3′). Afterward, the amplified products were cloned into the pGEMT-T easy vector (Promega, Madison, WI), and then DNA sequencing was performed on 10 individual clones (Macrogen, Seoul, Korea).

Sphere forming assay

Cells were grown in serum-free DMEM/F12 (Gibco) supplemented with B27 (Gibco), N2 (Gibco), 20 ng/mL basic fibroblast- (Peprotech, London, UK) and 20 ng/mL epidermal growth factor (Peprotech) onto 24-well ultra low attachment plates at 300 cells per well for 7 or 14 days, and then the size and number of spheres were determined using a phase-contrast Nikon microscope (TS100; Tokyo, Japan). To measure the size of sphere, 12 spheres per group were randomly selected.

Fluorescence-activated cell sorting

The cells were detached from the dishes with trypsin-EDTA (Gibco), counted, and washed in PBS (phosphate-buffered saline) containing 0.1% BSA (Bovine serum albumin) (Santa Cruz Biotechnology Inc.). 1×10^7 cells in 500 µl PBS containing 0.5% BSA were incubated with APC-conjugated fluorescence-labeled mouse anti-human CD133 (Miltenti Biotec, Bergisch Gladbach, Germany) or APC-conjugated isotype control mouse IgG2b (BD Biosciences) at 4 ℃ for 30 min in the dark. After washing, the labelled cells were sorted by flow cytometry, FACSAria™ (Special order system, BD Biosciences) into $CD133^+$ and $CD133^-$ cells.

Supplementary Figure S1.

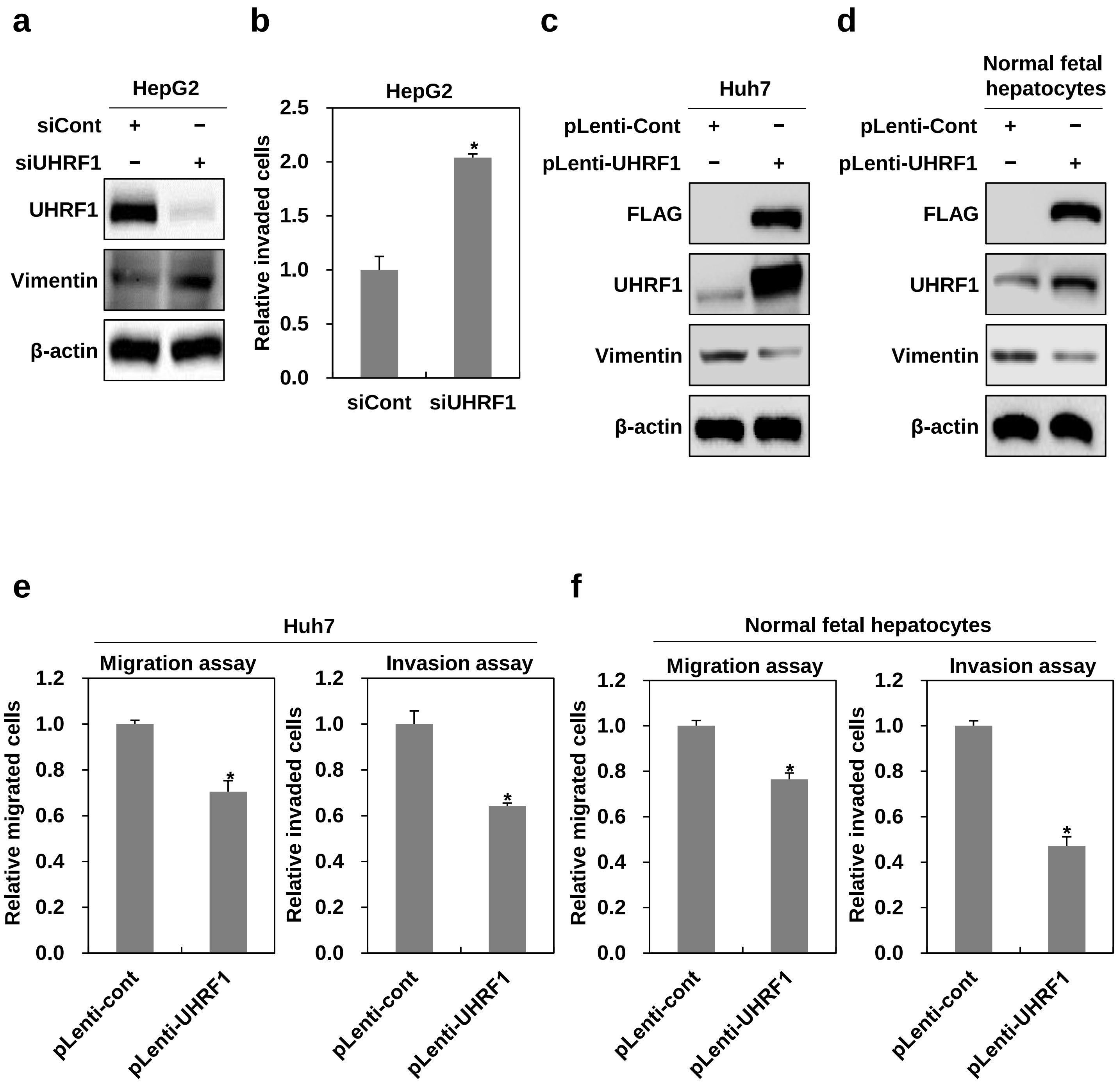

Supplementary Figure S1. continued

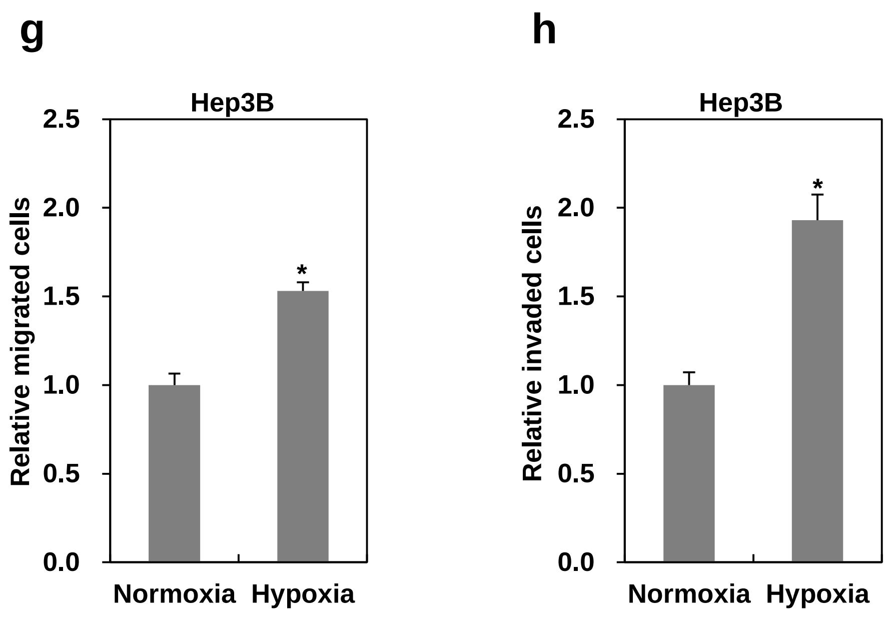

Supplementary Figure S2.

a

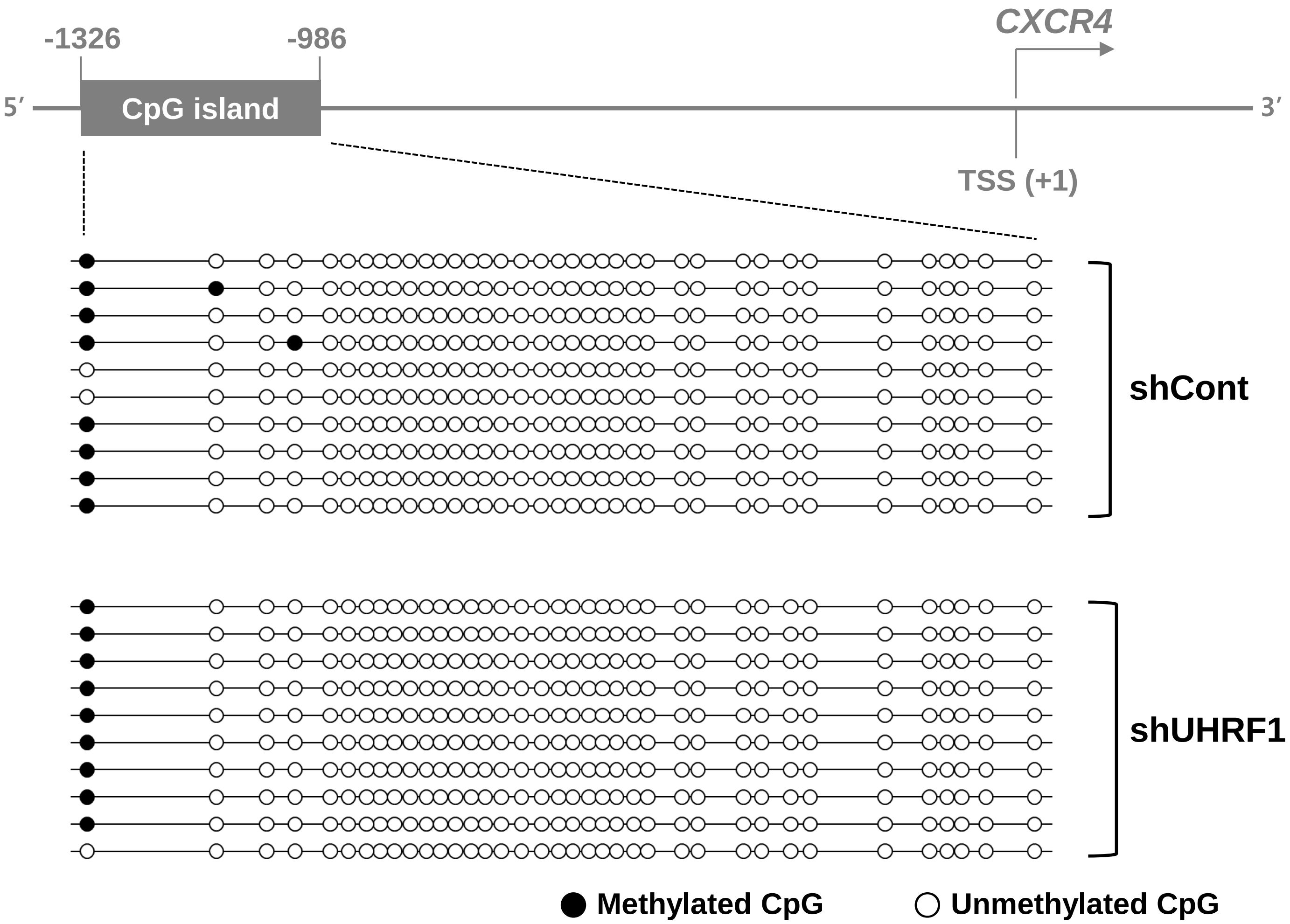

b

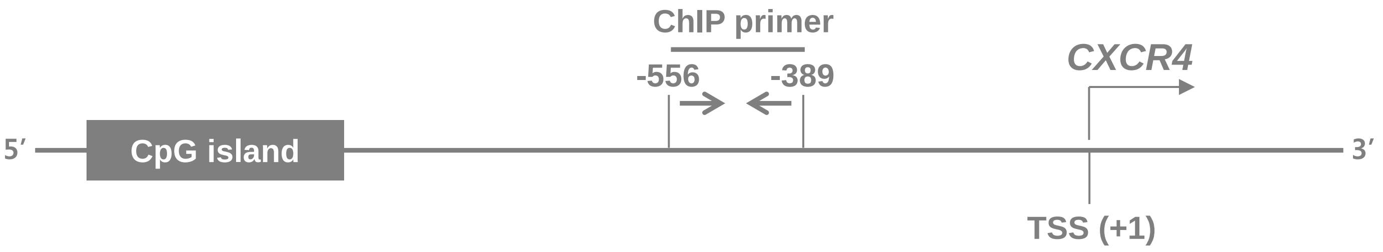

Supplementary Figure S3.

a

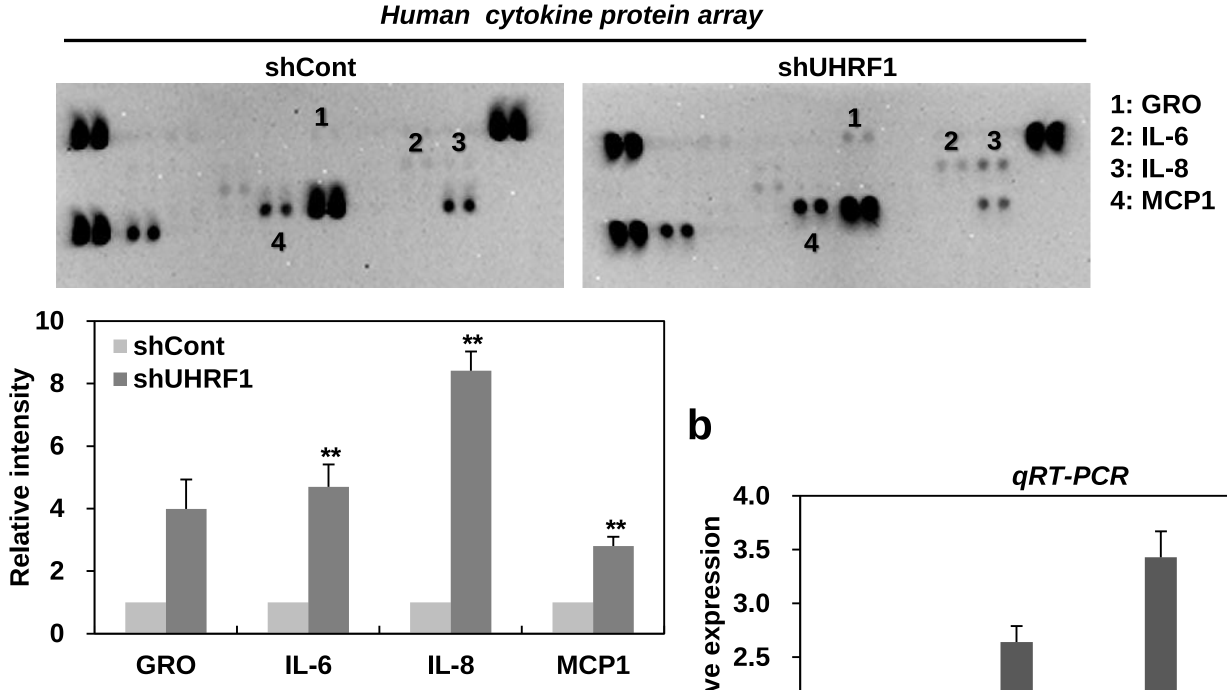

b

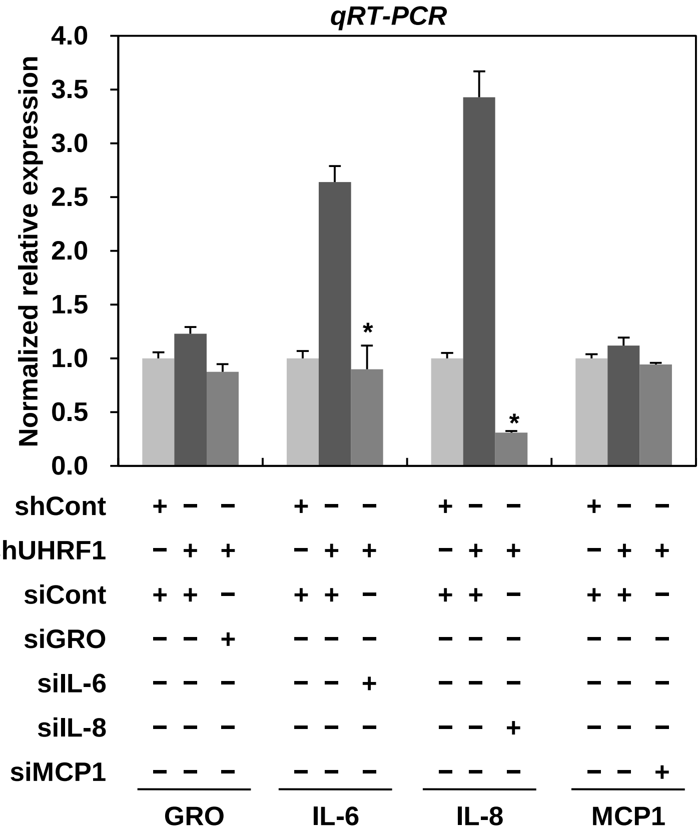

c

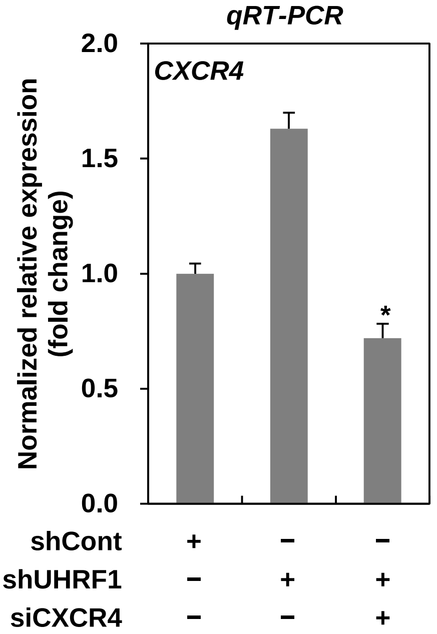

d

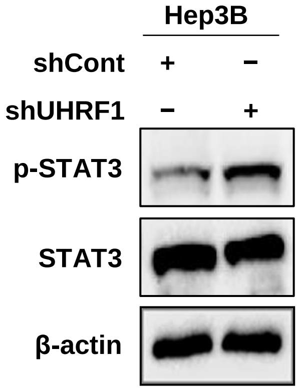

Supplementary Figure S4.

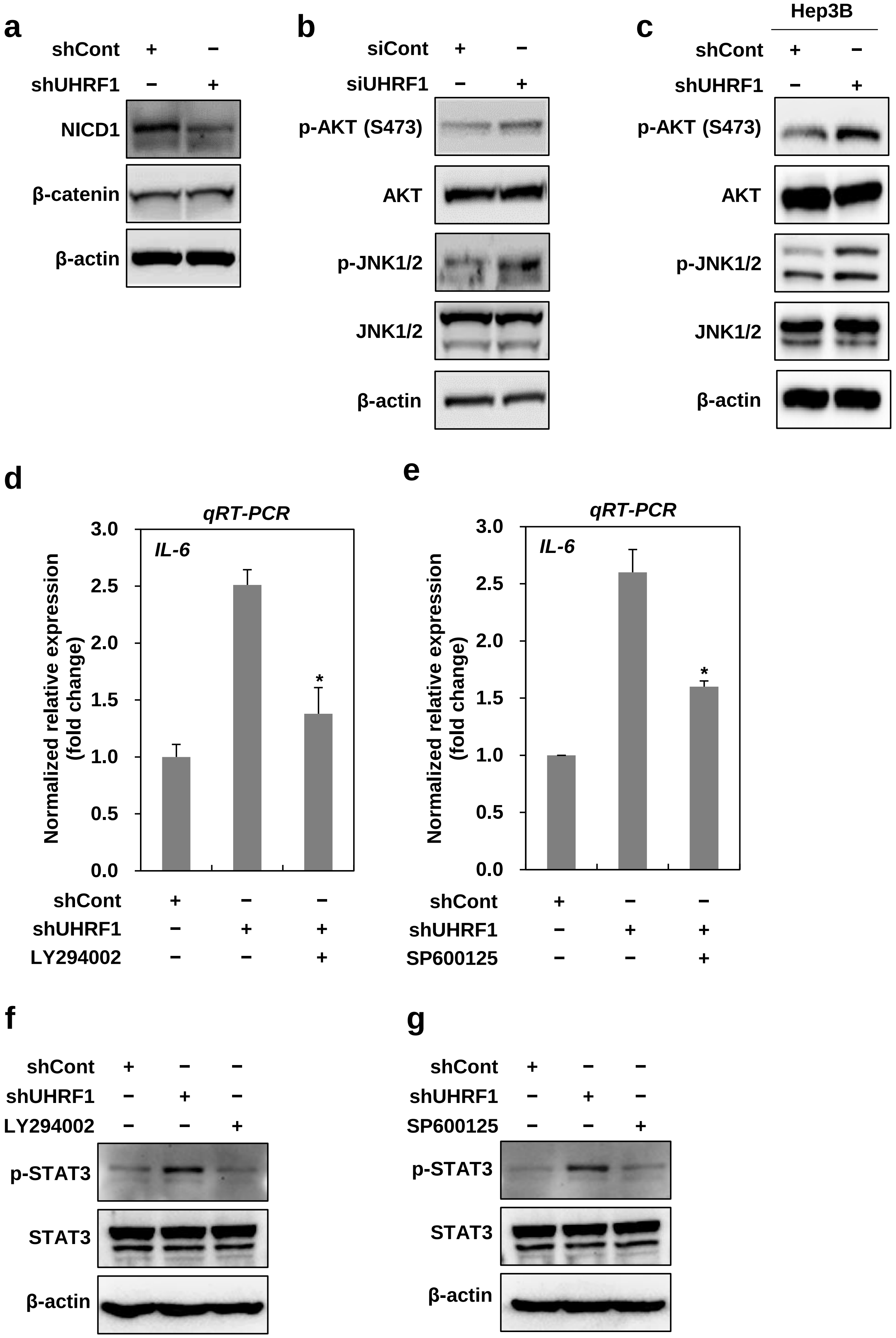

Supplementary Figure S4. continued

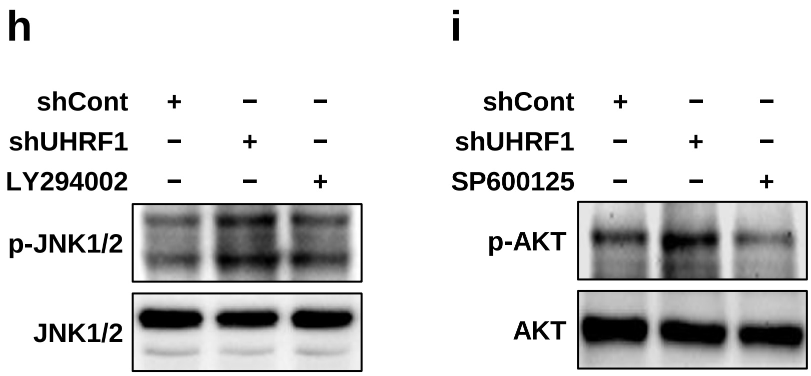

Supplementary Figure S5.

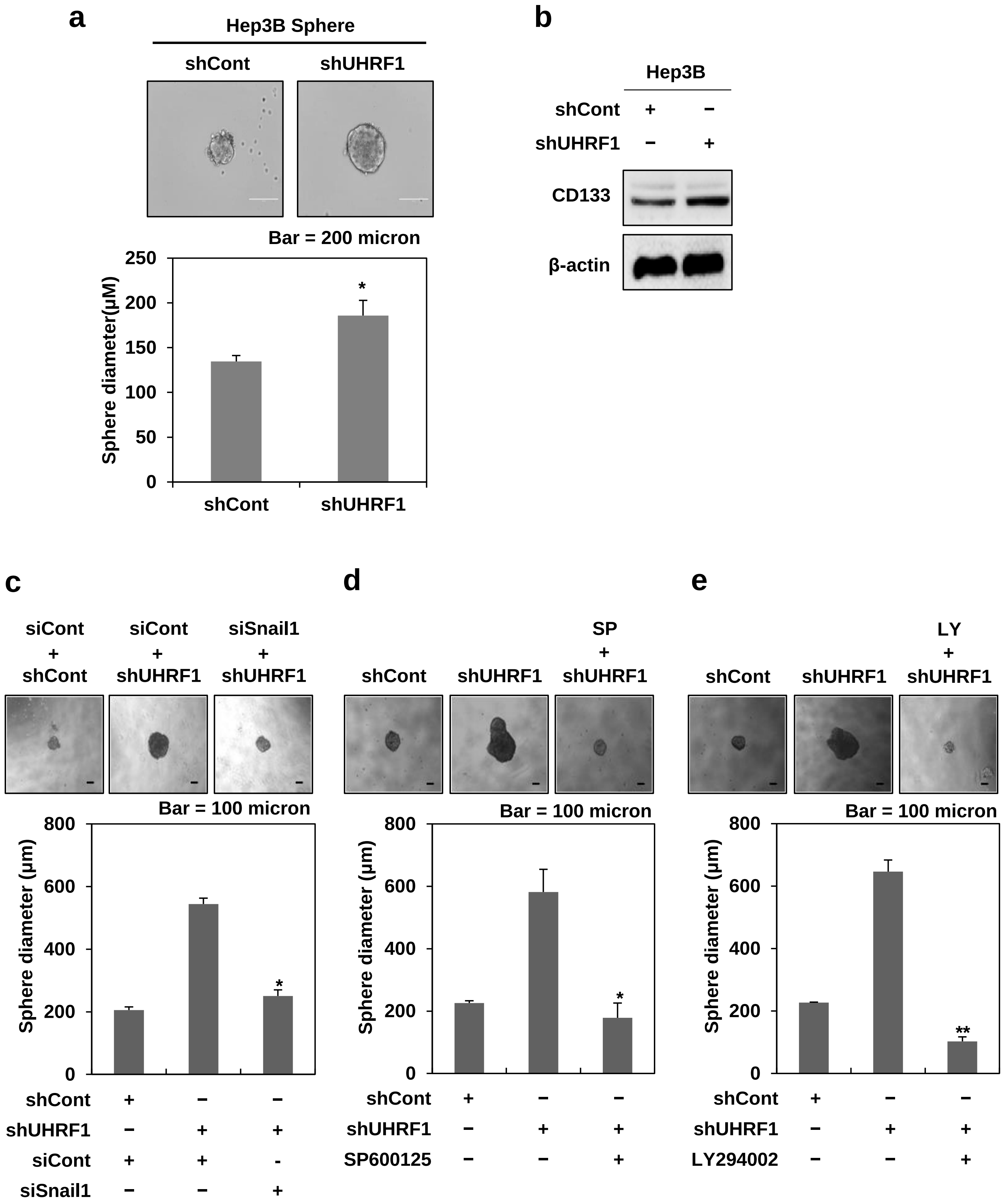

Supplementary Figure S6.

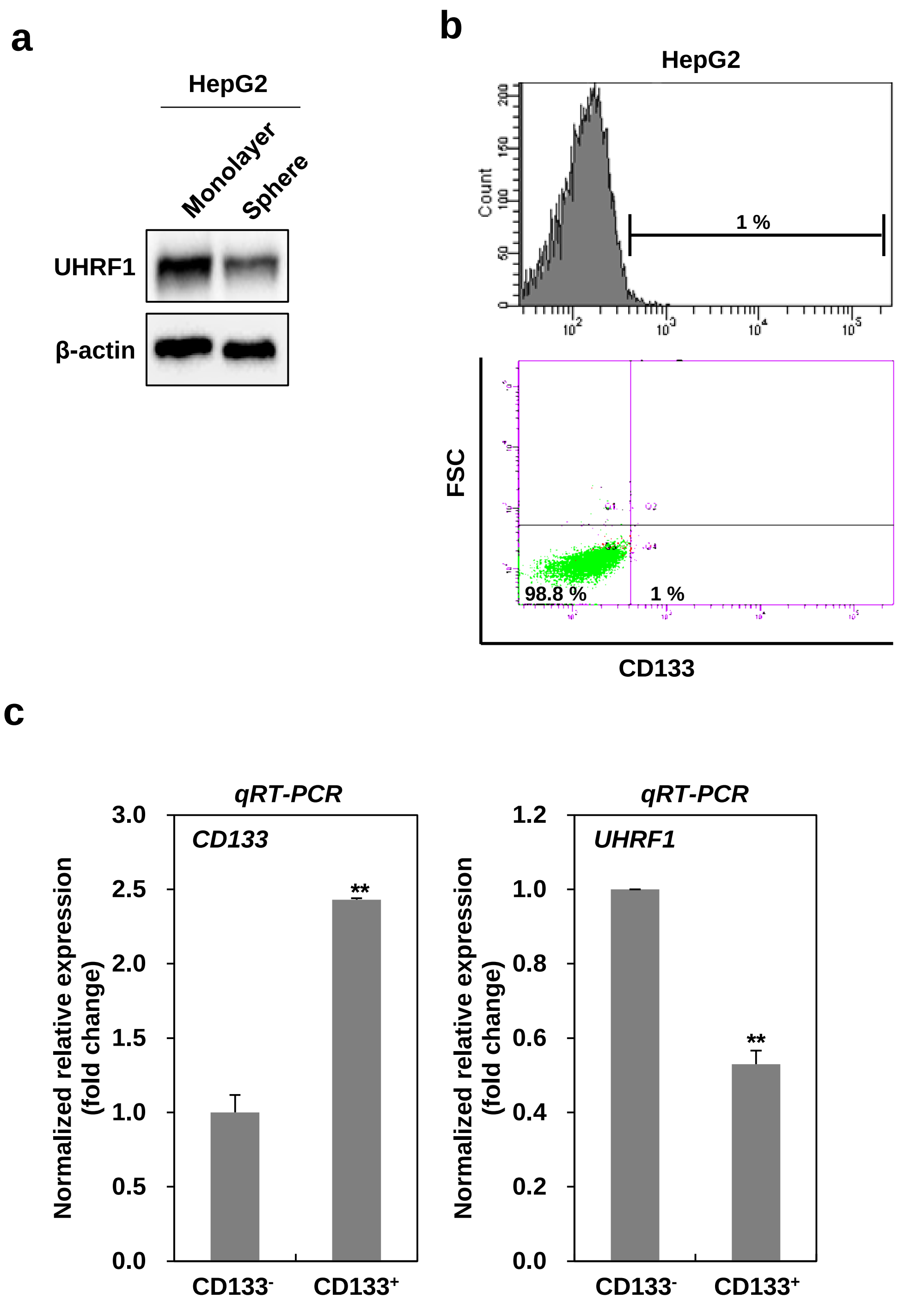

Supplementary Table S7.

List of Antibodies used in this study

Antibody	Company	Catalog #	Location
Anti-UHRF1 (ICBP 90)	Millipore	MABE308	Darmstadt, Germany
Anti-vimentin	Santa Cruz Biotechnology, Inc.	Sc-6260	Santa Cruz, CA, USA
Anti-E-cadherin	Santa Cruz Biotechnology, Inc.	Sc-8426	Santa Cruz, CA, USA
Anti-snail	Santa Cruz Biotechnology, Inc.	Sc-28199	Santa Cruz, CA, USA
Anti-p300	Santa Cruz Biotechnology, Inc	Sc-584	Santa Cruz, CA, USA
Anti-H3K27ac	Abcam	Ab4729	Cambridge, UK
Anti-H3K9me	Abcam	Ab8898	Cambridge, UK
Anti-STAT3	Santa Cruz Biotechnology, Inc.	Sc-7179	Santa Cruz, CA, USA
Anti-Sox2	Santa Cruz Biotechnology, Inc.	Sc-20088	Santa Cruz, CA, USA
Anti-p-AKT (S473)	Cell Signaling Technology	#9271	Danvers, MA, USA
Anti-AKT	Cell Signaling Technology	#9272	Danvers, MA, USA
Anti-p-ERK1/2	Cell Signaling Technology	#9101	Danvers, MA, USA
Anti-ERK1/2	Cell Signaling Technology	#9102	Danvers, MA, USA
Anti-p-JNK1/2	Cell Signaling Technology	#9255	Danvers, MA, USA
Anti-JNK1/2	Cell Signaling Technology	#9252	Danvers, MA, USA
Anti-p38	Cell Signaling Technology	#9212	Danvers, MA, USA
Anti-p-p38	Cell Signaling Technology	#9215	Danvers, MA, USA
Anti-p-STAT3 (Tyr705)	Cell Signaling Technology	#9145	Danvers, MA, USA
Anti-NICD1	Cell Signaling Technology	#4147	Danvers, MA, USA
Anti-HIF1α	Novus Biologicals, Inc.	NB100-479	Littleton, CO, USA
Anti-CD133	Novus Biologicals, Inc.	NB120-16518	Littleton, CO, USA
Anti-Oct4	BD Biosciences	611203	San Jose, CA, USA
Anti-β-catenin	BD Biosciences	610154	San Jose, CA, USA
Anti-N-cadherin	BD Biosciences	610920	San Jose, CA, USA
Anti-DDK (FLAG)	Sigma-Aldrich	F3165	St Louis, MO, USA
Anti-β-actin	Sigma-Aldrich	A5441	St Louis, MO, USA
Anti-hIL-6-IgG (IL-6 neutralizing antibody)	InvivoGen	Maba-hil6	San Diego, CA, USA
Horseradish peroxidase-conjugated anti-rabbit IgG	Thermo Scientific	G21234	Waltham, MA
Horseradish peroxidase-conjugated anti-mouse IgG	Thermo Scientific	G21040	Waltham, MA
TEXAS RED-conjugated goat anti-rabbit IgG	Vector Laboratories	TI-1000	Burlingame, CA, USA
TEXAS RED -conjugated goat anti-mouse IgG antibody	Vector Laboratories	TI-2000	Burlingame, CA, USA

Supplementary Table S8.

List of primers used in this study

Gene name	Forward	Reverse	Usage
CXCR4	5′-GTTACCATGGAGGGGATCAG-3′	5′-AACAAAAGGGCACTGAGACG-3′	qRT-PCR
CXCR4 promoter	5′-CTCGGTCCCAGCTATCTCC-3′	5′-GACTGGCTGAGGTTTGCAGT-3′	qRT-PCR after ChIP
GRO	5′-GAAAGCTTGCCTCAATCCTG-3′	5′-CACCAGTGAGCTTCCTCCTC-3′	qRT-PCR
IL-6	5′-CAGCCCTGAGAAAGGAACAT-3′	5′-CACCAGGCAAGTCTCCTCAT-3′	qRT-PCR
IL-8	5′-GCAGAGGGTTGTGGAGAAGT-3′	5′-CCCTACAACAGACCCACACA-3′	qRT-PCR
MCP1	5′-CCCCAGTCACCTGCTGTTAT-3′	5′-AGATCTCCTTGGCCACAATG-3′	qRT-PCR
Snail	5′-GAAAGGCCTTCAACTGCAAA-3′	5′-TGACATCTGAGTGGGTCTGG-3′	qRT-PCR
CD133	5′-ACATGAAAAGACCTGGGGG-3′	5′-GATCTGGTGTCCCAGCATG-3′	qRT-PCR
ALDH	5′-GATGCTTACCTACCACGGC-3′	5′-GGGCACGAAGGCTCATCATT-3′	qRT-PCR
ABCG2	5′-AAACTTCTGCCCAGGACTCA -3′	5′-CACGTGATTCTTCCACAAGC-3′	qRT-PCR
CD90	5′-CGCTCTCCTGCTAACAGTCTT-3′	5′-CAGGCTGAACTCGTACTGGA-3′	qRT-PCR
KLF4	5′-GTGCCCCGACTAACCGTTG-3′	5′-GTCGTTGAACTCCTCGGTCT-3′	qRT-PCR
OCT4	5′-GAAGGATGTGGTCCGAGTGT-3′	5′-CTGAGAAAGGAGACCCAGCA-3′	qRT-PCR
Nanog	5′-CTGTCTCTCCTCTTCCTTCCTC-3′	5′-CGGGACCTTGTCTTCCTTTT-3′	qRT-PCR
SOX2	5′-AAAAACGAGGGAAATGGGAG-3′	5′-GTCATTTGCTGTGGGTGATG-3′	qRT-PCR
GAPDH	5′-GAGCCCCAGCCTTCTCCATG-3′	5′-GAAATCCCATCACCATCTTCCAGG-3′	qRT-PCR

Supplementary figure legends

Figure S1. A comparison of EMT in UHRF1-overexpressing and UHRF1-deficient HepG2 cells. (a) Western blot analysis for UHRF1 and vimentin in HepG2 cells grown in the presence or absence of siRNA targeting UHRF1 for 48 h. (b) Effect of siRNA targeting UHRF1 on the invasive property of HepG2 cells. The cells were grown in the presence or absence of siRNA targeting UHRF1 for 48 h, and the invasion assay was performed using the Transwell chamber. (c and d) Western blot analysis for FLAG, UHRF1 and vimentin in pLenti-UHRF1-Huh7 cells and –normal fetal hepatocytes. (e and f) Effects of UHRF1 overexpression on the migratory and invasive properties of Huh7 cells and normal fetal hepatocytes. Migration and invasion assay were performed using the Transwell chamber. (g and h) The increase in the migratory and invasive properties of Hep3B cells grown under hypoxia for 24 h. The migration and invasion assay were performed using the Transwell chamber. β-actin was used as a loading control. Results from three independent experiments are expressed as means ± SEMs. (* $P < 0.05$, ** $P < 0.01$).

Figure S2. Effect of UHRF1 deficiency on the patterns of DNA methylation of the CpG island in CXCR4 gene promoter. (a) Schematic representation of 36 CpG sites in the CXCR4 promoter region from -986 to -1326 in HepG2 cells. Methylation analysis was performed in 10 clones for each cell line. Each row of circles represents a single clone, and each circle indicates a single CpG site. Open circle indicates unmethylated cytosine; filled circle indicates methylated cytosine. (b) One region at the CXCR4 locus used for the ChIP analysis.

Figure S3. Influence of UHRF1 deficiency on the secretion of cytokines from HepG2 cells. (a) Comparative quantification of GRO, IL-6, IL-8 and MCP1 in the conditioned medium from shCont- and shUHRF1-HepG2 cells using a cytokine protein array kit. (b) qRT-PCR analysis for the mRNA expression levels of GRO, IL-6, IL-8 and MCP1 in the presence or absence of siRNAs targeting GRO, IL-6, IL-8 and MCP1 in shUHRF1-HepG2 cells. (c) qRT-PCR analysis for the mRNA expression level of CXCR4 in the presence or absence of siRNA targeting CXCR4 in shUHRF1-HepG2 cells. (d) Western blot analysis for phosphorylated STAT3 in shCont- and shUHRF1-Hep3B cells. β-actin was used as a loading control. Results from three independent experiments are expressed as means ± SEMs. (* $P < 0.05$, ** $P < 0.01$).

Figure S4. Cross-talk between AKT and JNK in UHRF1-deficient HepG2 cells. (a) Western blot analysis for NICD1 and β-catenin in shCont- and shUHRF1-HepG2 cells. (b) Western blot analysis for phosphorylated AKT and -JNK in shCont- and shUHRF1-Hep3B cells. (c) Western blot analysis for phosphorylated AKT and -JNK in HepG2 cells transiently transfected with siRNA targeting UHRF1. HepG2 cells were grown in the presence or absence of siRNA targeting UHRF1 for 48 h, and then western blot analysis was performed. (d and e) Effect of LY294002 or SP600125 on the mRNA expression level of IL-6 in shUHRF1-HepG2 cells. The cells were grown in the presence or absence of LY294002 (2 μM) or SP600125 (5 μM) for 48 h, and then qRT-PCR analysis was performed. (f) Effects of LY294002 on the protein expression level of phosphorylated STAT3 in shUHRF1-HepG2 cells. The cells were grown in the presence or absence of LY294002 (2 μM) for 48 h, and western blot analysis was performed. (g) Effects of SP600125 on the protein expression level of phosphorylated STAT3 in shUHRF1-HepG2 cells. The cells were grown in the presence or absence of SP600125 (5 μM) for 48 h, and western blot analysis was performed. (h) Effects of LY294002 on the protein expression level of phosphorylated JNK in shUHRF1-HepG2 cells. The cells were grown in the presence or absence of LY294002 (2 μM) for 48 h, and western blot analysis was performed. (i) Effects of SP600125 on the protein expression level of phosphorylated AKT in shUHRF1-HepG2 cells. The cells were grown in the presence or absence of SP600125 (5 μM) for 48 h, and western blot analysis was performed. β-actin was used as a loading control. Results from three independent experiments are expressed as means ± SEMs. (* $P < 0.05$, ** $P < 0.01$).

Figure S5. UHRF1 deficiency-induced activation of AKT and JNK contributes to an increase in the sphere-forming ability of HepG2 cells.

(a) Quantification of sphere-forming abilities of shCont- and shUHRF1-Hep3B cells. The cells were grown in DMEM/F12 supplemented with B27, N2, basic fibroblast- and epidermal growth factor onto 24-well ultra low attachment plates at 500 cells per well for 7 days, and the size of spheres were determined. To measure the size of sphere, 12 spheres per group were randomly selected. (b) Western blot analysis for CD133 in shCont- and shUHRF1-Hep3B cells. (c) Effect of siRNA targeting Snail1 on the sphere-forming ability of shUHRF1-HepG2 cells. The cells were grown in the presence or absence of siRNA targeting Snail1 for 48 h, and sphere-forming assay was performed for 7 days. (d and e) Effect of SP600125 or LY294002 on the sphere-forming ability of shUHRF1-HepG2 cells. The cells were grown in the presence or absence of SP600125 (5 μM) or LY294002 (2 μM) for 48 h, and sphere culture was performed for 7 days. To measure the size of sphere, 12 spheres per group were randomly selected. Results from three independent experiments are expressed as means ± SEMs. (* $P < 0.05$, ** $P < 0.01$)

Figure S6. UHRF1 is downregulated in sphere-forming- or $CD133^+$ HepG2 cells. (a) A comparison of the protein expression of UHRF1 in monolayered and sphere-forming HepG2 cells. Sphere culture of HepG2 cells was performed for 7 days, and the protein expression of UHRF1 in sphere-forming HepG2 cells was compared with that in monolayered HepG2 cells using western blot analysis. (b) The isolation of $CD133^-$ and $CD133^+$ cells from HepG2 cells. The dot plot is divided into four quadrants for $CD133^-$ cells (Q1+Q3) and $CD133^+$ cells (Q2+Q4) which were isolated by flow cytometry sorting. (c) A comparison of the mRNA expression level of UHRF1 in $CD133^-$ and $CD133^+$ cells. After isolation of $CD133^-$ and $CD133^+$ cells, qRT-PCR analysis was performed. β-actin was used as a loading control. Results from three independent experiments are expressed as means ± SEMs. (* $P < 0.05$, ** $P < 0.01$).
